# Phenological segregation suggests speciation by time in the planktonic diatom *Pseudo‐nitzschia allochrona* sp. nov.

**DOI:** 10.1002/ece3.9155

**Published:** 2022-08-04

**Authors:** Isabella Percopo, Maria Valeria Ruggiero, Diana Sarno, Lorenzo Longobardi, Rachele Rossi, Roberta Piredda, Adriana Zingone

**Affiliations:** ^1^ Research Infrastructures for Marine Biological Resources Department Stazione Zoologica Anton Dohrn Naples Italy; ^2^ Integrative Marine Ecology Department Stazione Zoologica Anton Dohrn Naples Italy; ^3^ Istituto Zooprofilattico Sperimentale del Mezzogiorno Portici Italy; ^4^ Present address: Department of Veterinary Medicine University of Bari Aldo Moro Valenzano, Bari Italy

**Keywords:** cryptic species, diatoms, eco‐evolutionary dynamics, long‐term ecological research (LTER), phenology

## Abstract

The processes leading to the emergence of new species are poorly understood in marine plankton, where weak physical barriers and homogeneous environmental conditions limit spatial and ecological segregation. Here, we combine molecular and ecological information from a long‐term time series and propose *Pseudo‐nitzschia allochrona*, a new cryptic planktonic diatom, as a possible case of speciation by temporal segregation. The new species differs in several genetic markers (18S, 28S and ITS rDNA fragments and *rbc*L) from its closest relatives, which are morphologically very similar or identical, and is reproductively isolated from its sibling species *P. arenysensis*. Data from a long‐term plankton time series show *P. allochrona* invariably occurring in summer–autumn in the Gulf of Naples, where its closely related species *P. arenysensis*, *P. delicatissima*, and *P. dolorosa* are instead found in winter–spring. Temperature and nutrients are the main factors associated with the occurrence of *P. allochrona*, which could have evolved in sympatry by switching its phenology and occupying a new ecological niche. This case of possible speciation by time shows the relevance of combining ecological time series with molecular information to shed light on the eco‐evolutionary dynamics of marine microorganisms.

## Graphical abstract

We describe a new cryptic species in the marine diatom genus *Pseudo‐nitzschia*, *P. allochrona*, based on different phylogenetic markers and with the support of mating incompatibility with the sibling species *P. arenysensis*. Compared with its closest congeneric species in the Gulf of Naples, *P. allochrona* occupies a distinct temporal niche, which suggests it may have evolved in sympatry by switching its phenology and occupying a new ecological niche.

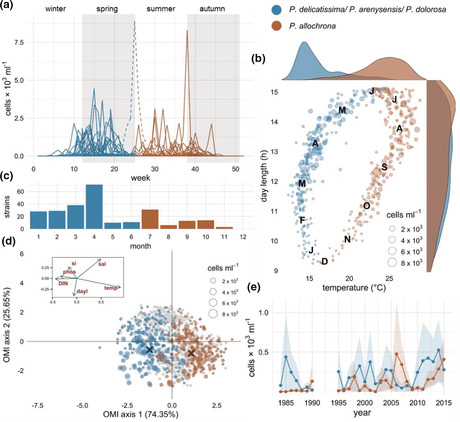

## 1 INTRODUCTION

The mechanisms underlying the emergence of new microalgal species in the marine realm are poorly explored. In the plankton, the diversity of such a relevant group of organisms has long been underestimated because of the scarcity of morphological features and the lack of adequate tools for the discrimination of meaningful units of ecology and evolution. The advent of molecular approaches in taxonomy and ecology has revolutionized our perception of microalgal diversity, revealing consistent genetic diversity coupled with morphological stasis in many instances and leading to an escalation in the discovery of cryptic or pseudocryptic species. The evidence of so far hidden diversity in marine microbes has increasingly emerged with the discovery of multiple species within iconic taxa long considered to be a single one. Notable examples are the diatoms *Skeletonema* (Sarno et al., 2005, 2007) and *Leptocylindrus* (Nanjappa et al., 2013) and the prasinophyte *Micromonas* (Simon et al., 2017). More evidences have been provided by massive sequencing of environmental DNA, which has revealed high level of interspecific and intraspecific genetic diversity in the microbial realm (de Vargas et al., 2015; Gaonkar et al., 2020; Moon‐van der Staay et al., 2001).

Distinct temporal or biogeographic patterns among pseudocryptic and cryptic species indicate that, in spite of morphological stasis, their phylogenetic diversity is also reflected in functional aspects such as their ecophysiological characteristics (Casteleyn et al., 2010; Foulon et al., 2008). At the same time, the discovery of the coexistence of many hardly distinguishable organisms in an apparently homogeneous environment exacerbates the so‐called “paradox of plankton” (Hutchinson, 1961), based on the idea that competitive exclusion in such a resource‐limited environment as the ocean should favor few fittest species occupying large, unstructured niches. At the global scale, genetic differences within taxa previously considered ubiquitous challenge the view of the prevalence of cosmopolitan microbes that would occur wherever the environment permits—“everything is everywhere, but, the environment selects” (Baas Becking, 1934; de Wit & Bouvier, 2006)—with limited biogeographic patterns and a consequent low diversity (Fenchel, 2005; Fenchel & Finlay, 2004).

The question left open by the findings of the last decades is how that great microbial diversity may arise at sea, and particularly in the plankton, where physical barriers are virtually absent and species' dispersal potential is unlimited. In these conditions, the role of geographic separation in promoting species diversification in allopatry appears unlikely (Palumbi, 1994). Sympatric speciation driven by ecological segregation (Potkamp & Fransen, 2019; Whittaker & Rynearson, 2017) could also be limited for planktonic microalgae in the often remixed photic zone. In the terrestrial habitat, the divergence of breeding times, that is, allochronic segregation, has been posited as a plausible mechanism for sympatric speciation, whereby phenological changes in part of the population can promote assortative mating and genetic divergence between subpopulations (Hendry & Day, 2005; Weis & Kossler, 2004). However, the possibility of sympatric speciation by allochronic segregation has rarely been considered for aquatic organisms (Rosser, 2016), although the existence of a temperature barrier for sexual reproduction between closely related species has been hypothesized to explain sexualization failure in some cases (Amato et al., 2007; Quijano‐Scheggia et al., 2009).

The pennate diatoms *Pseudo‐nitzschia* are needle‐like, chain‐forming planktonic organisms thriving in coastal waters around the world's seas. Their life cycle includes heterothallic sexual reproduction, through which these species re‐establish maximal cell size (Montresor et al., 2016). The diversity of the genus, now including 58 species (Guiry & Guiry, 2022), has expanded over the years due to the raised attention to the production in some species of neurotoxins (domoic acid, DA) that cause a syndrome known as amnesic shellfish poisoning. The use of molecular markers has much contributed to the description of new species within complexes of taxa that are hardly distinguishable morphologically, not even with electron microscopy (see Lim et al., 2018 for an updated review of the genus). These cryptic and pseudocryptic species may show distinct geographic ranges and temporal patterns (Bates et al., 2018; Ruggiero et al., 2015), as well as different biochemical and functional traits (Lamari et al., 2013), while intricate phylogenetic relationships and intraspecific genetic variations over the years indicate complex microevolutionary dynamics (D'Alelio & Ruggiero, 2015).

In the Gulf of Naples (GoN), 12 *Pseudo‐nitzschia* species are recorded (Ruggiero et al., 2015; Zingone et al., 2006). Among them, the *P. delicatissima*‐complex (Lundholm et al., 2006) is the most represented group, with three different species only identifiable with certainty by means of molecular methods: *P. delicatissima* sensu stricto, *P. arenysensis* and *P. dolorosa*. Here, we describe another cryptic *P. delicatissima*‐like species as *P. allochrona* sp. nov. based on sequences of diagnostic nuclear (18S rDNA, 28S rDNA, and ITS rDNA) and chloroplast (partial RUBISCO, *rbc*L) markers, ITS2 secondary structure and interbreeding experiments with its sibling species *P. arenysensis*. By coupling molecular information with environmental data from a 30 ys‐long time series, we build on the phenological and ecological peculiarities of *P. allochrona* to discuss possible mechanisms of speciation in the plankton realm.

## 2 MATERIALS AND METHODS

### 2.1 Samples and cultures

Sixty‐two strains of *Pseudo‐nitzschia allochrona* sp. nov. were isolated from surface waters of the Gulf of Naples (Mediterranean Sea) from 2007 to 2016, mainly from the Long Term Ecological Research Station MareChiara (LTER‐MC, 40°48.5′N, 14°15′E, depth ca 75 m; Ribera d'Alcalà et al., 2004; Zingone et al., 2019; Table S1). Two additional strains were isolated from the Ionian Sea (Mediterranean Sea) in September 2008. In addition, in this study, we considered 187 strains of other *P. delicatissima*‐like species obtained from the Gulf of Naples, which had been identified as *P. arenysensis*, *P. delicatissima* or *P. dolorosa* with molecular analyses in previous studies (Amato et al., 2007; Barra et al., 2013; Orsini et al., 2004), for which we could track the isolation date. Two further strains of *P. arenysensis* from the Gulf of Naples (BB16 and CM63, courtesy of M. Ferrante, SZN) were used in mating experiments. All strains were isolated by hand pipetting. Cultures were grown in F/2 medium and maintained at 20°C under an irradiance of 70–80 μmol photon m^−2^ s^−1^ and a 12:12 light:dark regime.

### 2.2 Microscopy

Live culture material of *P. allochrona* was observed and cell measurements taken under a Zeiss Axiophot and an Axiovert 200 light microscopes (Carl Zeiss). Pictures were taken with a Zeiss Axiocam digital camera (Carl Zeiss). For transmission electron microscopy (TEM) observations, clean diatom frustules were obtained boiling culture material for a few seconds with nitric (65%) and sulfuric (98%) acids (1:1:4, sample:HNO_3_:H_2_SO_4_) to remove organic matter and washing it with distilled water (modified from Round et al., 1990). The material was then mounted on Formvar‐coated grids and observed with a Philips 400 TEM (Philips Electron Optics BV). For scanning electron microscopy (SEM), material from successful mating experiments was fixed with glutaraldehyde (final concentration 2.5% v/v), placed on a filter in a Sweenex filter holder and dehydrated in a graded ethanol series (30%–100%). Filters were critical‐point dried, mounted on stubs, sputter‐coated with gold–palladium and observed with a JEOL JSM‐6500F SEM (JEOL‐USA Inc.).

### 2.3 Toxin analysis

Cultures of *P. allochrona* strains SZN‐B495 and SZN‐B524 were grown in 1 L Erlenmeyer flasks (20°C, irradiance 70–80 μmol photon m^−2^ s^−1^ and 12:12 light:dark regime), harvested at their late exponential growth phase and centrifuged (750 *g* for 10 min). The cell pellet was stored at −18°C until analysis. Cells were lysed by sonication for 5 min, then added with 500 μl of MeOH/H_2_O (1:1) mix, and vortexed for 3 min. Cells were lysed by sonication again for 5 min and centrifuged at 1700 *g* for 5 min. The clear supernatant was transferred into a glass test tube, and the pellets were resuspended in 500 μl of MeOH/H_2_O (1:1) and vortexed for 3 min. All steps were repeated three times. Then, the supernatant was evaporated and the residue resuspended with 500 μl of MeOH/H_2_O (1:1). This solution was centrifuged at 7690 *g* for 5 min and finally 5 μl were analyzed by LC–MS/TOF. Certified standard of domoic acid (DA) was purchased from the National Research Council of Canada (NRCC). Acetonitrile, methanol, and water were HPLC grade. Trifluoroacetic acid was obtained from VWR International (USA). The LC consisted of an Agilent 1100 instruments equipped with binary pump and an autosampler. Phenomenex Luna 3 μ PFP(2) (150 × 2.00 mm) was used for chromatographic separation. The isocratic mobile phase consisted of a mixture of 0.02% aqueous trifluoroacetic acid and acetonitrile in the ratio 90:10 (v/v), isocratic elution of 10% B at 0–15 min. The flow rate was 0.2 ml min^−1^. Sample solutions (8, 4, 2, and 0.4 ppm) were prepared in ACN/W (1:9), and 5 μl was injected. The MS/TOF analysis worked in positive ion mode, and mass range was set at *m/z* 100–1000 u at a resolving power of 10,000. The conditions of ESI source were as follows: drying gas (N_2_) flow rate, 11 ml min^−1^; drying gas temperature, 350°C; nebulizer, 45 psig; capillary voltage, 4000 V; fragmentor 225 V; skimmer voltage, 60 V. All the acquisition and analysis of data were controlled by Agilent LC–MS TOF Software (Agilent). Tuning mix (G1969‐85001) was used for lock mass calibration in our assay. Under these conditions, major peaks of DA would appear as the protonated ion at *m/z* 312, being accompanied by minor peaks consisting of sodium‐binding ions at *m/z* 334. For the reference DA material, LOD is 0.001 μg kg^−1^ (1 ng) and LOQ 0.01 μg kg^−1^ (10 ng).

### 2.4 Molecular analyses

Exponentially growing cultures were filtered on 0.8 μm pore size Isopore membrane filters (Millipore). Genomic DNA was extracted using the CTAB buffer as in (Tesson et al., 2011) and used as a template for the amplification of the following loci: partial 18S rDNA using Euk‐A and Euk‐B primers (Medlin et al., 1988); hypervariable (D1/D2) 28S rDNA region using DIR and D3Ca primers (Orsini et al., 2002); ITS rDNA using ITS‐1 and ITS‐4 primers (White et al., 1990); partial RUBISCO (*rb*cL) using *rbc*L1 and *rbc*L7 primers (Amato et al., 2007). Details of analyses carried out on individual strains are found in Table S1. PCRs were carried out in a PTC‐200 Peltier Thermal Cycler (MJ Research) using reaction conditions as in the above‐cited references for each of the amplified loci. The amplified fragments were purified using a QIAquick PCR purification kit (Qiagen Genomics) following the manufacturer's instructions, sequenced with the BigDye Terminator Cycle Sequencing technology (Applied Biosystems) and analyzed on an Automated Capillary Electrophoresis Sequencer “3730 DNA Analyzer” (Applied Biosystems).

*Pseudo‐nitzschia* sequences retrieved from GenBank for each marker (Table S2) were aligned with sequences of *P. allochrona* using MAFFT (Katoh & Standley, 2013), with the L‐INS‐i option. *Cylindrotheca fusiformis* and *Cylindrotheca* sp. were used as outgroup for 28S and *rbc*L, respectively, whereas *Fragilariopsis curta* and *F. cylindrus* were used as outgroups for 18S. Because the ITS region is highly variable, no outgroups were included in the analysis, in order to avoid ambiguous positions in the alignment. Maximum‐likelihood (ML) was used for all markers. The substitution model used for each marker was selected through the Bayesian information criterion (BIC) and Akaike information criterion (AIC) implemented in MEGA X (Kumar et al., 2018). Details of the phylogenetic parameters used per each marker can be found in the legend of Figure S1. ML analyses were performed in MEGA X and trees were built with 1000 bootstrap replicates. All the phylogenetic trees were visualized using the interactive online tool iTOL (https://itol.embl.de, Letunic & Bork, 2019).

The analysis of the net evolutionary divergence, that is, the number of base substitutions per site between ITS sequences of *P. allochrona* and of the most closely related species, was conducted in MEGA XP. Standard error estimates were obtained through a bootstrapping procedure with 1000 replicates.

The ITS2 secondary structure was predicted for sequences from strains SZN‐B509 (Gulf of Naples) and SZN‐B495 (Ionian Sea) for *P. allochrona* and NerD1 for *P. arenysensis* using RNA structure (Reuter & Mathews, 2010) with suboptimal structure parameters set as follows: maximum% energy difference 10, maximum number of structures 20, and window size 5. Format conversion (CT format to dot‐bracket format) was performed with RNApdbee (Antczak et al., 2014) and the 2D structures were drawn with VARNAv3.9 (Darty et al., 2009). The helices were labeled according to Mai and Coleman (1997). Compensatory base changes (CBCs) detection was performed with 4SALE v1.7 (Seibel et al., 2008), and hemi‐compensatory base changes (H‐CBCs) and other polymorphisms were observed manually.

### 2.5 Mating experiments

Sexual reproduction experiments were conducted on 18 exponentially growing cultures of *P. allochrona* isolated in July 2016 (Table S1). Prior to the experiments, the apical axis of 20 cells of each strain was measured in the light microscope (LM). One hundred and fifty‐three couples of strains were mixed at concentrations of about 2000 cells per ml, each couple in a well of six‐well culture plates containing 4 ml of F/2 medium, which were incubated at the conditions described above. The mixed cultures were examined daily using Zeiss Axiovert 200 light microscopes (Carl Zeiss). The content of some wells where sexual reproduction was taking place was fixed and prepared for SEM observations. Following a convention, the female mating type (−) was attributed to the strains that produced non‐motile gametes, which were recognized as gametangia holding the zygote. By crossing strains with different cell sizes, it was possible to identify the zygote‐bearing strain from its size and consider it as “female.” This allowed to identify as “female mating type” all other strains sexually incompatible with that one and as male mating types (+) the remainder.

Sexual compatibility was tested by crossing two strains of opposite mating type (BB16 and CM63) of *P. arenysensis* between them and with each of four strains of *P. allochrona* (MC1028‐B5, 8C3, MC1028‐C5, and MC1029A2) as described above.

### 2.6 Ecological analysis

Cell densities of *P. delicatissima*‐like morphotypes were estimated in 1154 phytoplankton samples collected by Niskin bottles at surface at the LTER‐MC stations fortnightly from January 1984 through July 1991 and weekly from February 1995 to December 2015. Samples fixed with buffered 37% formaldehyde solution (1.6% final concentration) were examined and counted following the Utermöhl method (Edler & Elbrächter, 2010) with a Zeiss Axiovert 200 LM (Carl Zeiss). Simultaneous data for environmental variables (temperature, salinity, and nutrients) were collected and quality controlled as described in Sabia et al. (2019).

Based on the collection dates of more than 250 strains over 10 years and the recurring seasonal patterns observed (see results), *P. delicatissima*‐like morphs occurring in summer and autumn were arbitrarily assigned to *P. allochrona*, while those recorded in winter and spring were assigned to other *P. delicatissima*‐like species, which included *P. delicatissima* sensu stricto, *P. arenysensis*, and *P. dolorosa*. Samples collected in late spring–early summer 2014, during an anomalous bloom not attributable to either *P. allochrona* or the other species based on recurrent seasonality, were excluded from the niche analysis. The environmental niches of *P. allochrona* and the other *P. delicatissima*‐like morphs were explored with the co‐inertia analysis Outlying Mean Index (OMI; Doledec et al., 2000), which generates ordination axes that maximize the separation between species occurrences in a multivariate environmental space. The positions of the species in the environmental space, representing the deviation of the species from a theoretical ubiquitous species occurring under all environmental conditions, were compared with simulated values (1000 random permutations) under the null hypothesis that each species is uninfluenced by the environment. The environmental map was defined based on surface values of physical (temperature and salinity) and chemical parameters (dissolved inorganic nitrogen, silicates, and phosphates) and by photoperiod.

## 3 RESULTS

**FIGURE 1:**
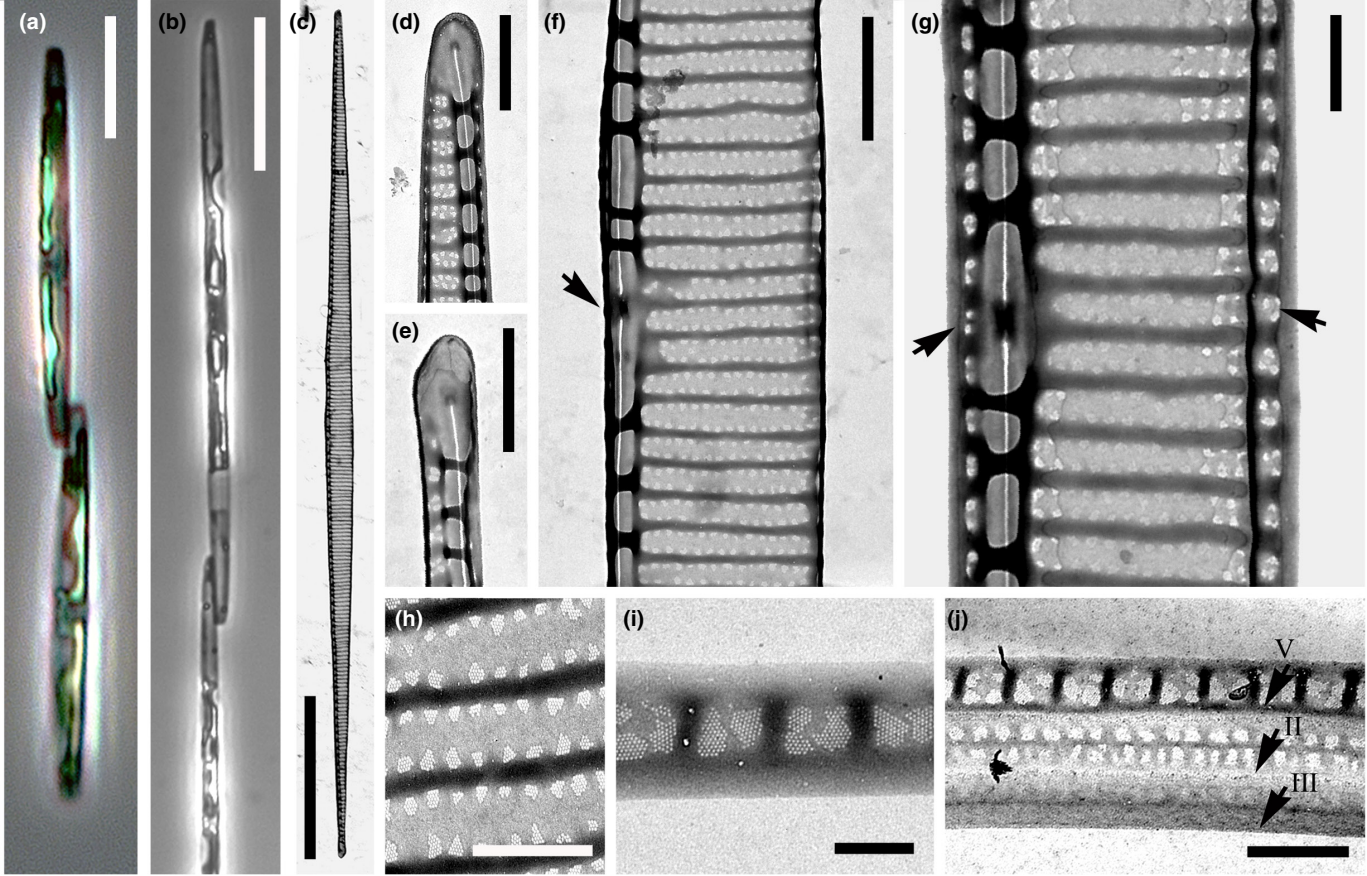
Morphology of *Pseudo‐nitzschia allochrona* sp. nov. LM (a, b) and TEM micrographs (c–j). (a) Cells in girdle view, strain MC784 4 II. (b) Long cells formed following sexual reproduction (cross of strains 9A2x9C3A, Table S3). girdle view. (c) Whole valve. (d) Valve end. (e) Valve end. (f) Central part of the valve face with central nodule. (g) Central part of the valve with central nodule and mantle (arrowheads). (h) Detail of the valve striae with two rows of pores typical of the *P. delicatissima‐*complex. (i) Detail of the valvocopula. (j) the three cingular bands, with arrows indicating their borders: V = valvocopula, II = second cingular band, III = third cingular band. (a): Strain MC784 4 II, (b): (c–j): Strain SZN‐B109. Scale bars: (a) = 5 μm, (b) = 20 μm, (c) = 10 μm, (d–f) = 1 μm, (g, h, j) = 0.5 μm, (i) = 0.2 μm.

*Pseudo‐nitzschia allochrona* Zingone, Percopo et Sarno sp. nov. (Figure 1a–j).

*Diagnosis*: Cells lanceolate with pointed ends forming stepped colonies. Apical axis 22–84 μm, transapical axis: 1.4–2.1 μm. Valves with larger central interspace, striae with two rows of irregular poroids, 10–12 in 1 μm. 20–26 fibulae and 34–44 interstriae in 10 μm. Cingulum with three open bands: (i) valvocopula, one‐two poroids high and two poroids wide, with 46–50 striae in 10 μm, (ii) second band, with a longitudinal silicified line flanked by two rows of poroids, and (iii) third band almost unperforated.

*Holotype*: Slide of strain SZN‐B501 deposited at the Museum of the Stazione Zoologica Anton Dohrn (SZN).

*Isotype*: Fixed material of SZN‐B501 deposited at the SZN Museum.

*Epitype*: Molecular characterization: sequences of 18S, 28S and ITS rDNA and *rbc*L of strain SZN‐B501 are deposited in GenBank with the following accession numbers: 18S, as KJ608076; 28S ON7755631; ITS, ON775460; *rbc*L, as KC801037.

*Type locality*: LTER‐MareChiara, 40°48′50″N; 14°15′0″E, Gulf of Naples (Mediterranean Sea) where the species generally occurs in summer–early autumn (*T*: 24–27.9°C), with strains occasionally isolated later in the year (*T* >18°C).

*Etymology*: the epithet (*allos*: other, *chronos*: time) refers to the distinct phenology of the species, that is, the time of the year when it is detected in the plankton, compared with other *Pseudo‐nitzschia delicatissima*‐like species that occur in the type locality.

*Pseudo‐nitzschia allochrona* belongs to the *P. delicatissima*‐complex (sensu Lundholm et al., 2006), which includes species with thin valves (generally <3 μm wide), with a central nodule and interstriae with two rows of poroids (Figure 1). In light microscopy, *P. allochrona* shows the typical features of several *P. delicatissima*‐like species, including thin valves, moderate length, and relative thickness of cell ends in lateral view, resulting in a pronounced step in cell chains (Figure 1a,b). In the electron microscope, the ultrastructure of *P. allochrona* frustules (Figure 1c–j) matches that of two cryptic species in the *P. delicatissima*‐complex, that is, *P. arenysensis* and *P. delicatissima*. The three species have widely overlapping density ranges for interstriae (ca. 34–45 in 10 μm), fibulae (ca. 19–26 in 10 μm), poroids (ca. 7–12 in 1 μm), and band striae (ca. 40–50 in 10 μm; Ajani et al., 2013; Lundholm et al., 2006; Quijano‐Scheggia et al., 2009, see Table A1 for detailed comparison of morphometric characters). Another species in the *P. delicatissima*‐complex, *P. dolorosa*, also found in the GoN, is also not distinguishable from *P. allochrona* in LM but has a lower density of interstriae, fibulae and poroids, and may have one or two rows of poroids (Lim et al., 2012; Lundholm et al., 2006). Among the other *P. delicatissima*‐like species, two were occasionally found in the Gulf of Naples by isolation or metabarcoding (Ruggiero et al., 2022). *P. micropora* is distinct from *P. allochrona* because it lacks a central nodule in the larger interspace between the two central fibulae (Ajani et al., 2013; Priisholm et al., 2002), while *P. decipiens* has a higher density of interstriae (41–47 in 10 μm) and band striae (48–55 in 10 μm; Lundholm et al., 2006; Teng et al., 2015).

**FIGURE 2:**
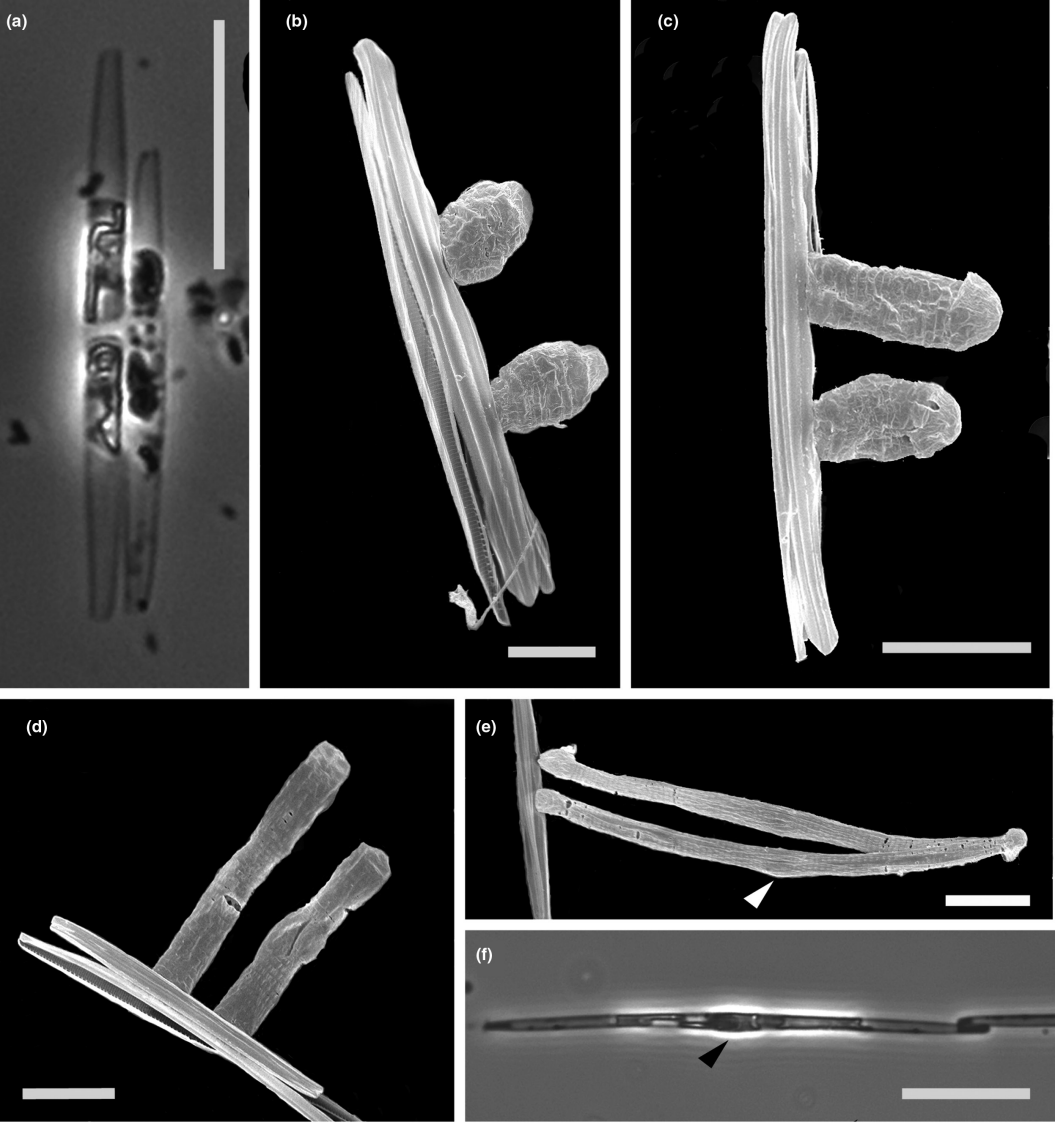
Life stages of *Pseudo‐nitzschia allochrona* sp. nov. during sexual reproduction. LM (a, f) and SEM (b–e). (a) Two paired gametangia of opposite mating types and different sizes, cross of strains 9A2x9C5. (c) Gametangia with two zygotes connected to the parental valve, cross 9B4x9C5. (c) Early auxospores, cross 9B4x9C5. (d) Elongated auxospores, cross 9B4x9C3a. (e) Mature auxospores with a bulge in the center (arrowheads), still connected to the parental valve, cross 9B4x9C5. (f) Long cell following the first divisions with a distinct central bulge (arrowhead), cross 9A2x9C5. Scale bars: (a and f) = 20 μm, (b) = 5 μm, (c–e) = 10 μm.

The size of the 18 strains of *P. allochrona* tested in breeding experiments ranged between 22 and 54 μm (38.5 ± 4.2 μm; *n* = 364) in apical axis. All strains mated in numerous successful crosses that allowed to identify two groups of 10 and 8 strains of opposite mating types (Table S3). Gametangia of different strains and at times of different size paired about 1 day after the inoculum (Figure 2a). Couples of zygotes, initially spherical (Figure 2b), modified synchronously into elongate auxospores (Figure 2c,d) attached to the frustule of one empty gametangium (the “female” one by convention). Mature auxospores were often curved, with a clear bulge in the center, a cap at each end and a transversal perizonium with fairly ornamented bands (Figure 2e). Initial cells (apical axis 72–84 μm, 79.9 ± 2.3 μm; *n* = 21) were also slightly curved (Figure 2f) but regained a straight shape after the first vegetative divisions (Figure 1b).

**FIGURE 3:**
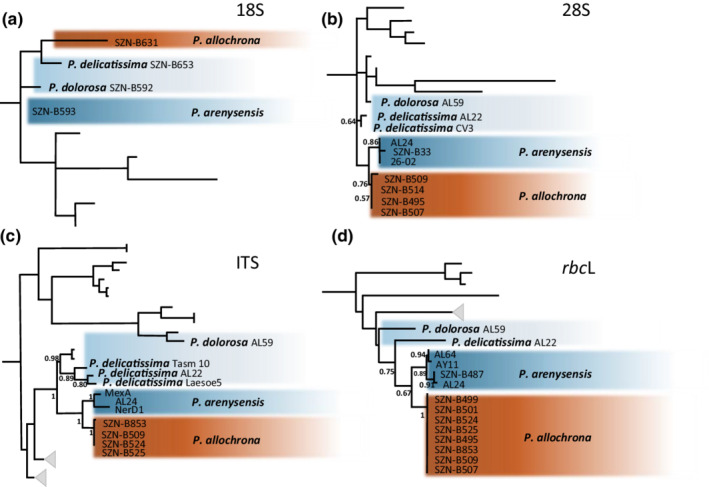
Maximum‐likelihood phylogenies of the *P. delicatissima*‐complex species living in sympatry in the Gulf of Naples. Excerpts from Figure S1 representing the complete phylogenetic trees. (a) 18S; (b) 28S; (c) ITS; and (d) *rbc*L. The new species *P. allochrona* is well separated in all markers from the closely related species that occur in the Gulf of Naples, namely, *P. delicatissima*, *P. dolorosa*, *and P. arenysensis*, and it is sister to *P. arenysensis* in all supported phylogenies, being closer to *P. delicatissima* only in the non‐supported 18S phylogeny.

All four markers investigated (18S, 28S, ITS, and *rbc*L) showed *P. allochrona* as distinct from all known congeneric species and clustering with moderate to high support with other *P. delicatissima*‐like species (Figure 3 and Figure S1). The new species was closely related to *P. arenysensis* in all phylogenies but the 18S one, where it was associated with *P. delicatissima* in a poorly supported clade. The net evolutionary divergence in ITS between *P. allochrona* and *P. arenysensis* (0.042 ± 0.010) was lower than that with *P. micropora* (0.082 ± 0.013) and *P. delicatissima* (0.090 ± 0.011; Table S4).

**FIGURE 4:**
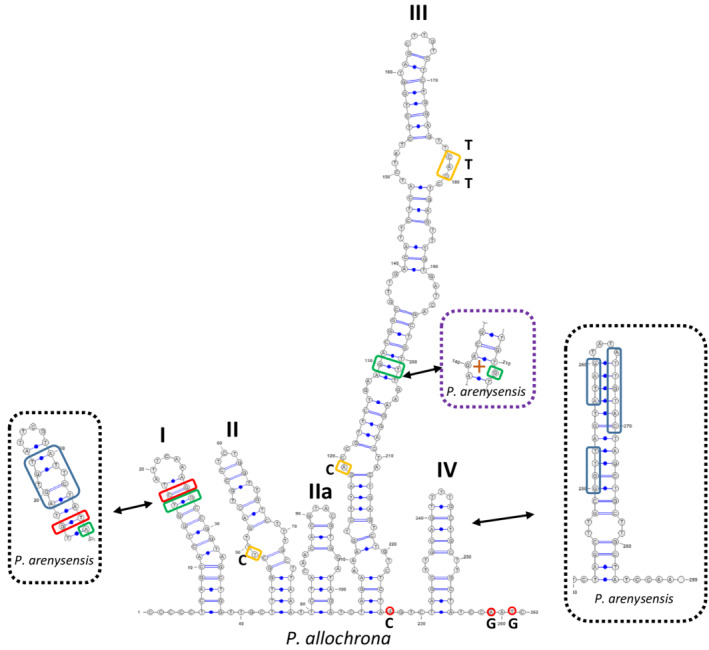
ITS2 secondary structure of *P. allochrona* (SZN‐B509) and comparison to that of the sibling species *P. arenysensis* (NerD1). Dashed black boxes: Indels in the alignment of the two species; red boxes: CBCs; green boxes: Hemi‐CBCs; yellow boxes: SNPs; and dashed purple box: A hemi‐CBC generating an internal pyrimidine‐pyrimidine loop (+). Red circles indicate intraspecific ITS polymorphisms between *P. allochrona* strains from the Gulf of Naples and those from the Ionian Sea (SZN‐B495).

The ITS2 secondary structure of *P. allochrona* showed four main helices (Helix I‐IV) and one pseudo‐helix (IIa), with a pyrimidine–pyrimidine mismatch in helix II, similar to other congeneric species (Amato et al., 2007; Figure 4). Compared with its closest relative *P. arenysensis*, *P. allochrona* ITS2 had one CBC in helix I (C‐G in *P. allochrona* ↔ G‐T in *P. arenysensis*) and two hemi‐CBCs in helices I (G‐T ↔ A‐T) and III (G‐T ↔ G‐G). In helix I, two deletions (AGTGT and ATTCT) determined the loss of one internal loop pyrimidine‐pyrimidine and a change in the hairpin loop structure. A single SNP was found in the internal loop (C → T) of helix II and four SNIPs in the internal loops (TTT → CAC and A → C) of helix III, where a deletion of GG produced the loss of an internal pyrimidine‐pyrimidine internal loop. The stem length of helix IV was shorter because of three deletions (GGTT, ATAG, and ATTGTAC), which also determined changes in the conformation of the hairpin loop.

Two interfertile *P. arenysensis* strains crossed with four *P. allochrona* strains of opposite mating type did not show any sign of sexual reproduction between the two species (Table S3).

Toxin analyses of strains SZN‐B524 and SZN‐B495 did not reveal the presence of domoic acid.

**FIGURE 5:**
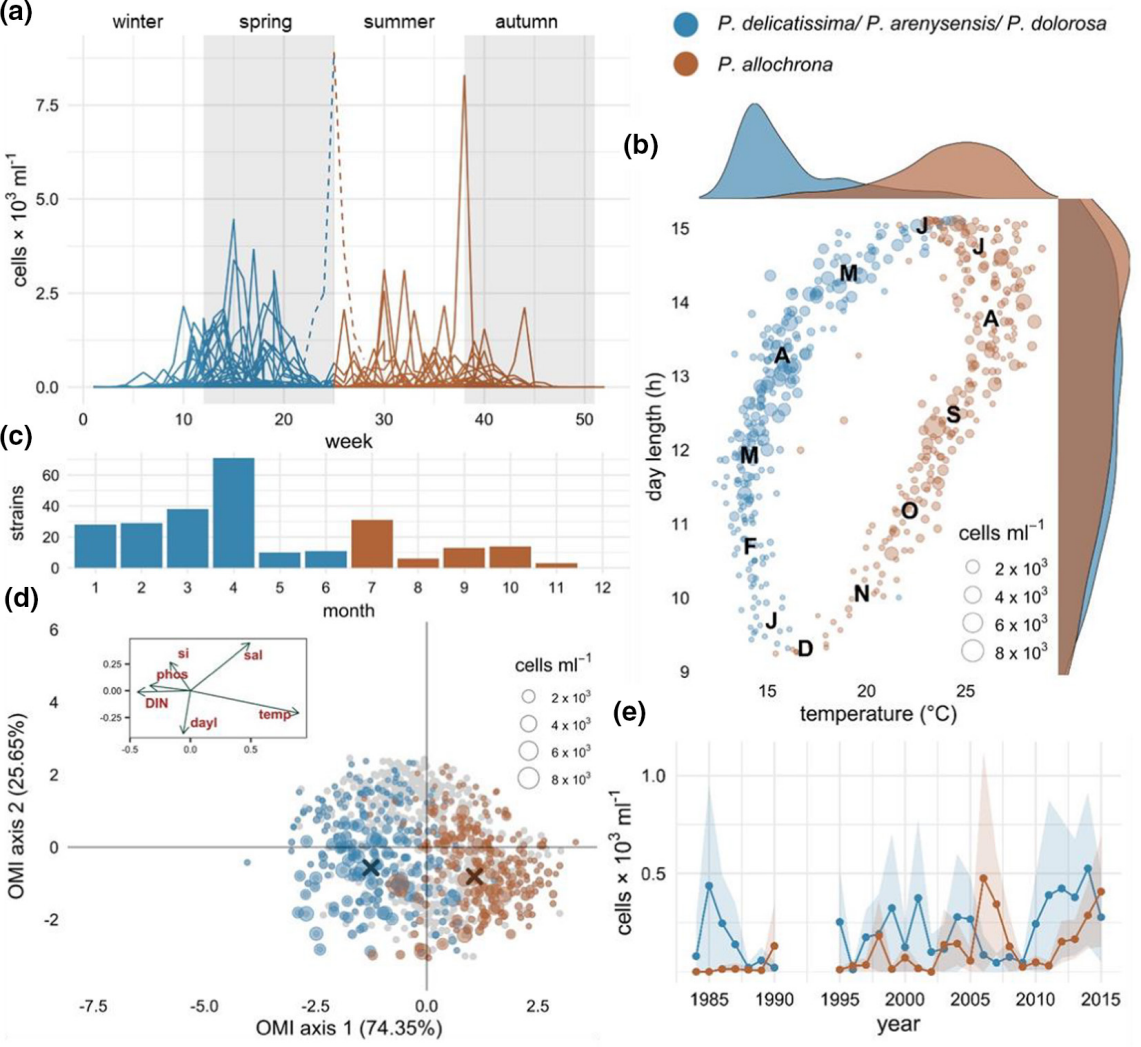
Distribution and ecology of the species of the *P. delicatissima*‐complex in the Gulf of Naples. (a) Annual distribution (1984–2015) of *P. allochrona* and other *P. delicatissima*‐like species (*P. delicatissima*, *P. arenysensis*, and *P. dolorosa*, lumped), light microscopy data. Lines represent different years. *P. Allochrona* was distinguished from its cryptic congeneric species based on its recurring occurrence in summer–autumn. The exceptional peak in late June–early July 2014 (dashed line) was not attributable to either species and hence was not included in the niche analysis of panel d. (b) Separation of *P. delicatissima*‐like species in the seasonal space identified by day length and temperature values. Letters are month names' initials. (c) Annual distribution of *P. allochrona* and the three other *P. delicatissima‐*like species living in the Gulf of Naples based on the isolation date (2004–2016) of 62 and 187 molecularly identified strains, respectively. (d) Niche analysis showing *P. allochrona* separated from the other congeneric species along the OMI axis 1, highly correlated with temperature (temp) and negatively correlated with nutrients (DIN): Dissolved inorganic nutrients; phos: phosphate; si: silicate. More and larger dots in the 3rd and 4th quadrants indicate higher frequency and abundance of all species with greater day length (day l) and lower salinity (Sal). Gray dots are samples with no *P. delicatissima*‐like species. (e) Interannual density variations (lines: annual average values; shadowed areas: CI 95%) of *P. delicatissima‐*like species at the LTER‐MC site in the Gulf of Naples.

At the LTER‐MC station, *P. delicatissima*‐like species usually showed a first bloom period from March through May and a second one from late June through mid‐September (Figure 5a,b), with minima generally in late spring and late autumn–winter but occasional peaks also in the latter periods. Through molecular analyses, all 62 *P. delicatissima*‐like strains isolated from the Gulf of Naples from late June onwards over 10 years resulted to be *P. allochrona*, while 187 strains isolated in the first part of the year over multiple years all belonged to *P. arenysensis* and *P. delicatissima*, and more rarely to *P. dolorosa* (Figure 5c). Based on this clear temporal separation in the isolation dates, and also supported by metabarcoding results over different years (see discussion), we arbitrarily assigned records of summer–autumn *P. delicatissima*‐like morphotypes in the LTER‐MC time series to *P. allochrona* and winter–spring ones to the remainder in order to assess the specificity of the ecological niche of the new species. A conspicuous bloom in a period of minima, in late spring–early summer 2014, could not be attributed confidently to either *P. allochrona* or the other species in lack of molecular data and was hence excluded from statistical analyses. Other possible overlaps between the two groups of *P. delicatissima*‐like morphs in the periods of their segregation (mid‐June–early July and late autumn–early winter) are deemed not to alter the results of niche analysis to a large extent, as those cases were rare or corresponded to periods of low abundances (Figure 5a). The first OMI axis in the niche analysis of all *P. delicatissima*‐morphs (Figure 5d) accounted for most of the variability (75.32%) and described the environmental gradient from early‐spring conditions, with relatively high nutrient levels and low temperature, to higher temperature and poorer nutrient concentrations in summer. The second OMI axis (27.68%) defined the environmental gradient of light and salinity separating the average habitat conditions occurring in late autumn and late spring. The niche positions of *P. delicatissima*‐like morphs significantly deviated from the origin of the multivariate space (*p*‐value <.001) pointing at the relationship of their seasonal distribution with environmental conditions. The clear separation along the first OMI axis reflected the association of *P. allochrona* with higher temperature in a relatively nutrient‐poor environment compared with the other *P. delicatissima*‐like morphs. In either conditions, longer days appeared to favor more numerous and intense blooms (Figure 5c,d). Over more than 30 years of sampling at the LTER‐MC site, densities of *P. delicatissima*‐like morphs occurring in summer–autumn, here attributed to *P. allochrona*, were null (1984–1985) or very low (1986–1989) in the first years of the time series. Peaks in the period 1991–1995 (not sampled) could have been missed but the abundance of *P. delicatissima*‐like morphs found in the second half of the year (presumably *P. allochrona*) increased particularly over the last 15 years, in some years overtaking that of the other species (Figure 5e).

## 4 DISCUSSION

Several results of this study support *P. allochrona* as a new cryptic species within the *P. delicatissima‐*complex. Morphologically undistinguishable by definition from some closely related species, namely *P. delicatissima* and *P. arenysensis*, its distinctiveness is clearly seen in the molecular signature of three nuclear and one chloroplast sequences that are commonly used for species delimitation in diatoms. Further, marked differences and conformational changes in the ITS secondary structure compared with its closest relative *P. arenysensis* and the failure of sexual reproduction experiments indicate mating incompatibility between the two sister species (Amato et al., 2007; Coleman, 2009).

The phenological signature represents a conspicuous character distinguishing *P. allochrona* from the other *P. delicatissima*‐like species living in sympatry in the Gulf of Naples. Since its first record in 2004 (as “*Pseudo‐nitzschia* new genotype”) in a clone library‐based DNA‐metabarcoding study of the 28S rDNA fragment (McDonald et al., 2007), *P. allochrona* has always been the only species of the group found in summer–autumn, never showing up among the more than 187 strains of the *P. delicatissima*‐complex retrieved in winter–spring in the area over more than 10 years, all invariably identified as *P. delicatissima*, *P. arenysensis*, or *P. dolorosa*. Temporal segregation is also confirmed by an annual overview based on 28S rDNA clone library, in which *P. allochrona* (as *P. delicatissima* IV) was responsible for the late summer‐early autumn blooms of 2009 and 2010 (Ruggiero et al., 2015), while a three‐year HTS study based on 17,763 environmental 18S rDNA‐V4 barcodes has again shown *P. allochrona* to be abundant in summer in 2011 and 2013 (Ruggiero et al., 2022). In all the three above‐mentioned metabarcoding studies, *P. arenysensis/P. dolorosa* (not separated by the V4‐18SrDNA marker) and *P. delicatissima* overlap to a large extent in their occurrences, being the main contributors to the spring peak of this species complex. Peaks of *P. arenysensis* in some years followed and in other years preceded those of *P. delicatissima*, which actually co‐occurred with *P. allochrona* in late June‐early July of 2013. Interestingly, detailed molecular investigations on individual *Pseudo‐nitzschia* haplotypes from the 3‐year HTS dataset revealed an intragenomic *P. allochrona* variant intermediate between the prevalent *P. allochrona* and *P. arenysensis* haplotypes, as well a small number of *P. allochrona* haplotypes in early winter (Ruggiero et al., 2022).

The phenological segregation between *P. allochrona* and its cryptic congeneric species matches the differences in environmental conditions among different periods of the year, whereby temperature mainly, and nutrient levels to some extent, seem to be the drivers of the separation of the two annual peaks of the species complex in the Gulf of Naples. While nutrients in this area should not be limiting in either seasonal context (Ribera d'Alcalà et al., 2004), the difference in ca. 10°C between spring and summer–autumn temperature values suggests ecophysiological variations between the species responsible for blooms at different times of the year. In *Thalassiosira rotula*, changes in the genetic structure among populations also correlate strongly with water temperature at the spatial and temporal scale (Whittaker & Rynearson, 2017), whereas a salinity gradient drives spatial patterns of genetic diversity in the case of *Skeletonema marinoi* in the Baltic Sea (Sjoqvist et al., 2015). The relationship between neutral and functional genetic diversity within and among species is complex (Orsini et al., 2013), but the adaptive response under new environmental condition can be very rapid in diatoms (Pargana et al., 2020; Schaum et al., 2018). In the picoprasinophyte *Ostreococcus*, the tiniest eukaryote, cryptic sister species occupy different temporal and spatial niches (Limardo et al., 2017) and also show profound functional genomic differences (Palenik et al., 2007). In our case, biochemical differences between *P. allochrona* (as *P*. cf. *delicatissima*) and two of its closely related species, *P. arenysensis* and *P. delicatissima*, have been found in lipoxygenase enzymes mediating the metabolism of eicosapentaenoic acid (Lamari et al., 2013). Differences between *P. arenysensis* and *P. delicatissima* have also been described at the whole transcriptome level (Di Dato et al., 2015), which altogether provide a first indication of functional differences within this group of cryptic species.

Morphological identity and relatively small phylogenetic distance between *P. arenysensis* and *P. allochrona*, along with differences in their ecological and temporal niches, rouse some speculations on possible modes of speciation. Based on the lack of detection, followed by low abundance values, of summer–autumn *P. delicatissima*‐morphs in the first years of the time series, *P. allochrona* could be a warm water species recently introduced in the Mediterranean Sea or in the Gulf of Naples, where it has found its optimal niche in summer. At least two other cases of sudden appearance have been recorded in the Gulf of Naples time series, namely *P. multistriata* in 1995 and *Skeletonema tropicum* in 2002 (Zenetos et al., 2010). However, differently from the latter cases, the area where *P. allochrona* could have been introduced from cannot be traced. Despite numerous and detailed studies focusing on the genus *Pseudo‐nitzschia* in the Mediterranean Sea and all over the world, so far *P. allochrona* has only been found in the Gulf of Naples (Lamari et al., 2013; McDonald et al., 2007; Ruggiero et al., 2015), Ionian Sea (this paper) and more recently in the Adriatic Sea (Arapov et al., 2020; Giulietti et al., 2021; Pugliese et al., 2017). In the latter area, *P. allochrona* (as *P*. cf. *arenysensis*) has been found in summer–autumn, like in the Gulf of Naples and Ionian Sea, while *P. arenysensis* has never been detected. The high spatial and taxonomic resolution offered by metabarcoding data could help clarifying the biogeography of these cryptic species, allowing to detect them in remote areas and thus supporting allopatric speciation, but the present distribution would hardly reflect the distribution at the time of speciation (Hendry, 2009). In addition, identical sequences may be shared by different taxa over the global scale for the usual marker for metabarcoding, the 18 s rDNA‐V4 region (Piganeau et al., 2011), thus requiring that species presence recorded by these approaches be confirmed by isolation/cultivation studies or the use of more variable markers.

Hence, allopatric speciation can hardly be demonstrated for these cryptic microorganisms. Although it cannot be ruled out either, as an alternative mechanism it is tempting to postulate that the separation of the sister species *P. allochrona* and *P. arenysensis* may have occurred in sympatry in the Gulf of Naples. Habitat heterogeneity promotes species diversity at both the ecological and evolutionary time scale, preventing competitive exclusion and providing new niches to be occupied by different, co‐existing species, eventually leading to ecological speciation (Chesson & Warner, 1981; Hutchinson, 1961). Whereas spatial partitioning of ecological conditions is hard to conceive in the planktonic realm, especially for microalgae confined to the photic zone, environmental factors can vary considerably along the year in strongly seasonal environments, such as the Mediterranean Sea, whereby time can replace space in creating the habitat heterogeneity required for ecological divergence, thus leading to allochronic speciation. A similar example of divergence by time in the same genus is offered by *Pseudo‐nitzschia galaxiae*, a species that blooms in the Gulf of Naples in three different periods of the year with populations of three size classes (Cerino et al., 2005). The size classes actually correspond to distinct ribotypes (McDonald, 2007) that are also retrieved as distinct in eDNA metabarcoding studies (Ruggiero et al., 2015). Whether the three *P. galaxiae* populations are separate species needs further investigation, but the coherent pattern observed in size ranges, genetics and timing of the blooms provides a further possible case of isolation by time and allochronic speciation processes.

Genetic divergence among populations occurring at different times has been observed in various microalgal groups (Lebret et al., 2012; Richlen et al., 2012; Rynearson et al., 2006; Sassenhagen et al., 2018; Tammilehto et al., 2016; Whittaker & Rynearson, 2017), suggesting that temporal segregation could be a general mechanism for speciation in marine protists. Yet, phenology, that is, the seasonal time window of species' occurrences, has rarely been considered a stable, endogenous character in phytoplankton, whereby the alternation of different species over the seasons is postulated to be strictly driven by changes in environmental conditions. Nevertheless, annually recurrent patterns are seen in long‐term analysis of nano‐ and microphytoplankton species of the Gulf of Naples, where photoperiod is the most important explanatory variable driving community turnover (Longobardi et al., 2022; Ribera d'Alcalà et al., 2004). Recent massive eDNA sequencing from other areas has also revealed recurrent occurrence of picoplanktonic taxa against marked environmental variability (Giner et al., 2019; Lambert et al., 2019), which can be explained based on endogenous rhythms and stability of phenological characteristics.

Population dynamics in *Pseudo‐nitzschia* species is compatible with a scenario of stable, endogenous phenological rhythms, because synchronous growth timing maximizes encounter probability, which is a key element for sexual reproduction of heterothallic species in a highly dispersive habitat. Sexual reproduction in natural *Pseudo‐nitzschia* populations has been inferred from shifts in cell size (D'Alelio et al., 2010) and rarely observed, but interestingly in September 2006 a massive sexual event was recorded at station LTER‐MC at the end of a bloom of a *P. delicatissima*‐like species (Sarno et al., 2010), which was probably *P. allochrona* based on the time of the year. As *Pseudo‐nitzschia* species are not known to produce benthic stages, sparse cells persist in the plankton outside the density peak time (Cipolletta et al., 2022) and occasionally may give rise to blooms in other periods of the year. In this context, slight variations in peak times and unusual blooms in some years, as the one recorded in June 2014, suggest that the attempt to colonize new time windows may occur frequently in this genus or in phytoplankton species at large, which could be seen as wandering across the seasons in search of new ecological niches.

The mechanisms underlying allochronic speciation, and speciation in general, are not easy to clarify (Orsini et al., 2013), as both neutral and selective processes could be involved. Time and environmental variables covary, making it difficult to discern their respective contribution to the patterns observed in this study. One possibility is that ecological segregation arises as a consequence of phenological shifts in the timing of the maximum abundance, a mechanism that would reduce competition for resources among individuals within a population (Devaux & Lande, 2008). While most individuals respond rapidly and similarly to the relevant environmental cues, phenological characters often follow a skewed distribution (Forrest & Miller‐Rushing, 2010), the long tail implying that a small part of the population may experience new environmental conditions during key life‐cycle steps, such as reproduction. In such conditions that promote assortative mating, reduced gene flow can eventually lead to reproductive isolation between groups (Hendry & Day, 2005). This situation is somewhat analogous to a founder effect, where a subsample of the population colonizes new niches, diverging from the mother population via genetic drift (Barton & Charlesworth, 1984). Phenological variations could be favored by the phenotypic plasticity that is typical of microalgae and/or by rapid adaptation to new ecological conditions (Schaum et al., 2018), or by selective processes acting on the standing intraspecific diversity (Orsini et al., 2013), which is also quite ample in phytoplankton populations (Rengefors et al., 2017).

Separation by time could be rapid enough as to be observable on decadal time scales, as no clear‐cut boundary exists between the timescales of ecological and evolutionary processes (Carroll et al., 2007; Hendry et al., 2007). Contemporary evolution could be traced especially in microbial organisms, which are characterized by high growth rates and a few to several tens of generations over a single bloom season. In fact, swift genetic variations may take place in phytoplankton (Collins et al., 2014; Rengefors et al., 2017), where interannual variations in the genetic structure were actually observed in another species of the genus, *P. multistriata* (Tesson et al., 2014) which showed intermittent periods of weak and strong intraspecific diversity over the same bloom season (Ruggiero et al., 2018).

Not different from terrestrial plants, unicellular aquatic phototrophs can capture information from light and possess genes that are involved in the regulation of biological rhythms at various scales (Annunziata et al., 2019; Fortunato et al., 2015). Circa‐annual rhythms and regular phenological patterns associated with photoperiodic response suggest that these microorganisms may also be able to measure the time of the year (Anderson & Keafer, 1987; Lambert et al., 2019). In this perspective, diversity in biological rhythms of plankton would be an optimal substrate for their evolutionary changes, contributing to isolation by time and speciation. In support of this proposition, future studies should address genetic variations in sympatric populations and sibling species over the seasonal and interannual timescale and assess their relationships with functional adaptation through the analysis of neutral and adaptive genetic variations, an approach now made possible by the accessibility of metagenomics and metatranscriptomic technologies. In this respect, the present study highlights the potential of combining molecular and ecological information over long‐term time series in order to trace the eco‐evolutionary dynamics and shed light on speciation mechanisms in the plankton.

## AUTHOR CONTRIBUTIONS

**Isabella Percopo:** Data curation (equal); formal analysis (equal); investigation (lead); resources (equal); validation (equal); visualization (equal); writing – original draft (lead); writing – review and editing (supporting). **Maria Valeria Ruggiero:** Data curation (equal); formal analysis (equal); investigation (lead); resources (equal); validation (equal); visualization (equal); writing – original draft (lead); writing – review and editing (supporting). **Diana Sarno:** Conceptualization (supporting); data curation (equal); investigation (supporting); project administration (supporting); resources (supporting); supervision (equal); validation (supporting); writing – review and editing (supporting). **Lorenzo Longobardi:** Data curation (equal); formal analysis (equal); investigation (equal); visualization (equal); writing – original draft (equal); writing – review and editing (supporting). **Rachele Rossi:** Formal analysis (equal); investigation (equal); writing – original draft (equal); writing – review and editing (supporting). **Roberta Piredda:** Formal analysis (equal); investigation (equal); visualization (equal); writing – original draft (equal); writing – review and editing (supporting). **Adriana Zingone:** Conceptualization (lead); data curation (equal); funding acquisition (lead); investigation (supporting); project administration (lead); resources (supporting); supervision (equal); validation (supporting); writing – original draft (supporting); writing – review and editing (lead).

## FUNDING INFORMATION

The research program LTER‐MC is funded by the Stazione Zoologica Anton Dohrn. The study was supported by the Italian RITMARE flagship Project, funded by MIUR under the NRP 2011‐2013, approved by the CIPE Resolution 2/2011 of 23.03.2011 (grant to IP), by the Italian project MIUR‐FIRB Biodiversitalia (RBAP10A2T4; grant to RP) and by the project PONDIV (PseudO‐Nitzschia: DIVersity behind an image, grant to MVR) funded by SZN.

## CONFLICT OF INTEREST

We declare no conflict of interest.

## Supporting information


Table S1

Table S2

Table S3

Table S4

Figure S1

## Data Availability

Molecular data produced in this study are available in GenBank. Abundance data of *Pseudo‐nitzschia delicatissima*‐like morphs and related environmental data used for niche analysis are available in the public repository Dryad at https://doi.org/10.5061/dryad.7h44j0zx7.

## APPENDIX A

**TABLE A1:** Morphometric data of *P. allochrona* and morphologically related species. Average and standard deviation in brackets, when available

	References	Valve shape	Width (μm)	Length (μm)	Central nodule	Striae in 10 μm	Fibulae in 10 μm	Poroids in 1 μm	Rows of poroids	Band striae in 10 μm
*P. allochrona* (as *P*. cf. *arenysensis*)	This study	Lanceolate	1.4–2.1 (1.8 ± 0.2)	32–84^a^	+	34–44 (40.3 ± 2.1)	20–26 (23.3 ± 1.5)	10–12 (10.7 ± 1.6)	2	46–50 (47.7 ± 1.5)
	Giulietti et al. (2021)	Linear	1.5–2.3 (1.9 ± 0.2)	29.1–50.6 (38.5 ± 5.2)	+	36–42 (38.3 ± 1.6)	16–36 (21.5 ± 2.2)	8–12 (10.1 ± 0.9)	2	42–52 (46.0 ± 2.3)
*P. arenysensis*	Quijano‐Scheggia et al. (2009)	Lanceolate	1.6–2.5	22–84^b^	+	34–43	20–26	7–12	2	40–50
	Ajani et al. (2013)	Lanceolate, symmetrical	1.8–2.7 (2.1 ± 0.2)	33.6–45.8 (39.8 ± 5.3)	+	38–45 (40.4 ± 1.5)	20–26 (22.8 ± 2.2)	8–11 (9.4 ± 0.9)	2	40–46 (42 ± 2.8)
*P. bucculenta*	Gai et al. (2018)	Lanceolate	2.7–3.6 (3.0 ± 0.3)	19–31 (24.9 ± 3.6)	+	28–35 (31.4 ± 1.7)	16–21 (18.4 ± 1.2)	5–7.5 (6.7 ± 0.6)	1–2	38–39 (38 ± 0.6)
*P. chiniana*	Huang et al. (2019)	Lanceolate	2.3–2.6 (2.4 ± 0.1)	42–58 (49.7 ± 1.5)	+	30–34 (32.8 ± 1.3)	17–22 (19.3 ± 1.8)	4–6 (5 ± 1)	1–2	38–40
*P. decipiens*	Lundholm et al. (2006)	Lanceolate	1.4–2.4 (1.9 ± 0.3)	29–64	+	41–46 (43.2 ± 1.2)	20–26 (24.0 ± 1.4)	9–13 (11.4 ± 1.2)	2	48–55 (51.8 ± 1.7)
	Teng et al. (2015)	Lanceolate, symmetrical	1.7–2.0	41.8–49.1	+	43–47	22–26	8–13	2	48–54
*P. delicatissima*	Lundholm et al. (2006)	Lanceolate	1.5–2.0 (1.8 ± 0.2)	19–94^a^	+	35–40 (36.8 ± 1.5)	19–26 (21.4 ± 1.6)	8–12 (10.1 ± 1.2)	2	43–48 (44. ± 1.6)
*P. dolorosa*	Lundholm et al. (2006)	Lanceolate, asymmetrical	2.5–3.0 (2.6 ± 0.2)	30–59	+	30–36 (34.5 ± 1.4)	18–22 (20.0 ± 1.0)	5–8 (6.6 ± 0.8)	1–2	40–44 (42.0 ± 1.4)
	Lim et al. (2012)	nd	1.8–2.1 (2.0 ± 0.2)	42.4–43.0 (42.7 ± 0.3)	+	35–37 (36.2 ± 0.8)	21–22 (21.6 ± 0.5)	5–6 (5.8 ± 0.5)	1	nd
*P. hainanensis*	Chen et al. (2021)	Lanceolate	1.8–2.0	23–45	+	30–34	17–22	4–8	2	41–42
*P. hallegraeffii*	Ajani et al. (2018)	Lanceolate, asymmetrical	1.9–3.1	25.6–55.4	+	34–40	16–22	6–8	1–2	43–56
*P. micropora*	Priisholm et al. (2002)	Lanceolate	1.3–2.0	31–57	‐	41–46	21–29	9–12	2	48–54
	Ajani et al. (2013)	Lanceolate, symmetrical	1.8–2.3 (2.0 ± 0.1)	33.1–36.0 (34.9 ± 0.9)	‐	42–50 (45.9 ± 2.9)	23–30 (27.1 ± 2.7)	9–13	2	54–60 (59.3 ± 2.1)
*P. prolongatoides*	Almandoz et al. (2008)	Lanceolate	1.5–2.6	20–85	+	29–33	16–21	10–13	2–3	nd
*P. sabit*	Teng et al. (2015)	Falcate, asymmetrical	1.4–2.6	22.5–69.0^a^	+	38–45	17–26	7–13	2	50–58
*P. turgiduloides*	Almandoz et al. (2008)	Lanceolate	2–2.9	81–126	+	18–24	10–14	7–10	1–2	nd
*P. turgidula*	Almandoz et al. (2008)	Lanceolate	2.3–2.5	41–79	+	24–28	15–18	7–9	2–3	nd
*P. yuensis*	Dong et al. (2020)	Lanceolate	1.8–2.5	37–44	+	38–43	19–24	6–7	1–2	43–48

Abbreviation: nd, not determined.

^a^ Maximum length measured on the initial cell.

^b^ Maximum length from the initial cell length from Amato et al. (2005, as *P. delicatissima*).

## References

[ece39155-bib-0001] Ajani, P. , Murray, S. , Hallegraeff, G. , Lundholm, N. , Gillings, M. , Brett, S. , & Armand, L. (2013). The diatom genus *Pseudo‐nitzschia* (Bacillariophyceae) in New South Wales, Australia: Morphotaxonomy, molecular phylogeny, toxicity, and distribution. Journal of Phycology, 49(4), 765–785. 10.1111/jpy.12087 27007209

[ece39155-bib-0002] Ajani, P. A. , Verma, A. , Lassudrie, M. , Doblin, M. A. , & Murray, S. A. (2018). A new diatom species *P. hallegraeffii* sp. nov. belonging to the toxic genus *Pseudo‐nitzschia* (Bacillariophyceae) from the east Australian current. PLoS One, 13, e0195622. 29649303 10.1371/journal.pone.0195622 PMC5896966

[ece39155-bib-0003] Almandoz, G. O. , Ferreyra, G. A. , Schloss, I. R. , Dogliotti, A. I. , Rupolo, V. , Paparazzo, F. E. , Esteves, J. L. , & Ferrario, M. E. (2008). Distribution and ecology of *Pseudo‐nitzschia* species (Bacillariophyceae) in surface waters of the Weddell Sea (Antarctica). Polar Biology, 31, 429–442.

[ece39155-bib-0004] Amato, A. , Kooistra, W. H. C. F. , Levialdi Ghiron, J. H. , Mann, D. G. , Pröschold, T. , & Montresor, M. (2007). Reproductive isolation among sympatric cryptic species in marine diatoms. Protist, 158, 193–207. 10.1016/j.protis.2006.10.001 17145201

[ece39155-bib-0005] Amato, A. , Orsini, L. , D'Alelio, D. , & Montresor, M. (2005). Life cycle, size reduction patterns, and ultrastructure of the pennate planktonic diatom *Pseudo‐nitzschia delicatissima* (Bacillariophyceae). Journal of Phycology, 41, 542–556.

[ece39155-bib-0006] Anderson, D. M. , & Keafer, B. A. (1987). An endogenous annual clock in the toxic marine dinoflagellate *Gonyaulax tamarensis* . Nature, 325(6105), 616–617. 10.1038/325616a0 3808064

[ece39155-bib-0007] Annunziata, R. , Ritter, A. , Fortunato, A. E. , Manzotti, A. , Cheminant‐Navarro, S. , Agier, N. , Huysman, M. J. J. , Winge, P. , Bones, A. M. , Bouget, F. Y. , Cosentino Lagomarsino, M. , Bouly, J. P. , & Falciatore, A. (2019). bHLH‐PAS protein RITMO1 regulates diel biological rhythms in the marine diatom *Phaeodactylum tricornutum* . Proceedings of the National Academy of Sciences of the United States of America, 116(26), 13137–13142. 10.1073/pnas.1819660116 31171659 PMC6600994

[ece39155-bib-0008] Antczak, M. , Zok T , Popenda M , Lukasiak P , Adamiak RW , Blazewicz J , & Szachniuk M . (2014). RNApdbee‐a webserver to derive secondary structures from pdb files of knotted and unknotted RNAs. Nucleic Acids Research, 42(Web Server issue), 368–372. doi:10.1093/nar/gku330 PMC4086112 24771339

[ece39155-bib-0009] Arapov, J. , Bužančić, M. , Penna, A. , Casabianca, S. , Capellacci, S. , Andreoni, F. , Skejić, S. , Bakrač, A. , Straka, M. , Mandić, J. J. , & Ninčević Gladan, Ž. (2020). High proliferation of *Pseudo‐nitzschia* cf. *arenysensis* in the Adriatic Sea: Ecological and morphological characterisation. Mediterranean Marine Science, 21(3), 16. 10.12681/mms.22932

[ece39155-bib-0010] Baas Becking, L. (1934). Geobiologie of inleiding tot de milieukunde the Hague. W.P. Van Stockum & Zoon.

[ece39155-bib-0011] Barra, L. , Ruggiero, M. V. , Sarno, D. , Montresor, M. , & Kooistra, W. C. H. F. (2013). Strengths and weaknesses of microarray approaches to detect *Pseudo‐nitzschia* species in the field. Environmental Science and Pollution Research, 20(10), 6705–6718. 10.1007/s11356-012-1330-1 24065245

[ece39155-bib-0012] Barton, N. H. , & Charlesworth, B. (1984). Genetic revolutions, founder effects, and speciation. Annual Review of Ecology and Systematics, 15(1), 133–164. 10.1146/annurev.es.15.110184.001025

[ece39155-bib-0013] Bates, S. S. , Hubbard, K. A. , Lundholm, N. , Montresor, M. , & Leaw, C. P. (2018). *Pseudo‐nitzschia*, *Nitzschia*, and domoic acid: New research since 2011. Harmful Algae, 79, 3–43. 10.1016/j.hal.2018.06.001 30420013

[ece39155-bib-0014] Carroll, S. P. , Hendry, A. P. , Reznick, D. N. , & Fox, C. W. (2007). Evolution on ecological time‐scales. Functional Ecology, 21(3), 387–393. 10.1111/j.1365-2435.2007.01289.x

[ece39155-bib-0015] Casteleyn, G. , Leliaert, F. , Backeljau, T. , Debeer, A. E. , Kotaki, Y. , Rhodes, L. , Lundholm, N. , Sabbe, K. , & Vyverman, W. (2010). Limits to gene flow in a cosmopolitan marine planktonic diatom. Proceedings of the National Academy of Sciences, 107(29), 12952–12957. 10.1073/pnas.1001380107 PMC2919969 20615950

[ece39155-bib-0016] Cerino, F. , Orsini, L. , Sarno, D. , Dell'Aversano, C. , Tartaglione, L. , & Zingone, A. (2005). The alternation of different morphotypes in the seasonal cycle of the toxic diatom *Pseudo‐nitzschia galaxiae* . Harmful Algae, 4(1), 33–48. 10.1016/j.hal.2003.10.005

[ece39155-bib-0017] Chen, X. M. , Pang, J. X. , & Huang, C. X. (2021). Two new and toxigenic *Pseudo‐nitzschia* species (Bacillariophyceae) from Chinese southeast coastal waters. Journal of Phycology, 57, 335–344. 33174223 10.1111/jpy.13101

[ece39155-bib-0018] Chesson, P. L. , & Warner, R. R. (1981). Environmental variability promotes coexistence in lottery competitive systems. The American Naturalist, 117(6), 923–943.

[ece39155-bib-0019] Cipolletta, F. , Russo, A. , D'Alelio, D. , Margiotta, F. , Sarno, D. , Zingone, A. , & Montresor, M. (2022). Vertical distribution of *Pseudo‐nitzschia* in the Gulf of Naples across the seasons. Mediterranean Marine Science, 23(3), 525–535. 10.12681/mms.28147

[ece39155-bib-0020] Coleman, A. W. (2009). Is there a molecular key to the level of “biological species” in eukaryotes? A DNA guide. Molecular Phylogenetics and Evolution, 50(1), 197–203. 10.1016/j.ympev.2008.10.008 18992828

[ece39155-bib-0021] Collins, S. , Rost, B. , & Rynearson, T. A. (2014). Evolutionary potential of marine phytoplankton under ocean acidification. Evolutionary Applications, 7(1), 140–155. 10.1111/eva.12120 24454553 PMC3894903

[ece39155-bib-0022] D'Alelio, D. , Ribera d'Alcalà, M. , Dubroca, L. , Sarno, D. , Zingone, A. , & Montresor, M. (2010). The time for sex: A biennial life cycle in a marine planktonic diatom. Limnology and Oceanography, 55, 106–114. 10.4319/lo.2010.55.1.0106

[ece39155-bib-0023] D'Alelio, D. , & Ruggiero, M. V. (2015). Interspecific plastidial recombination in the diatom genus *P* *seudo‐nitzschia* . Journal of Phycology, 51(6), 1024–1028. 10.1111/jpy.12350 26986997

[ece39155-bib-0024] Darty, K. , Denise, A. , & Ponty, Y. (2009). VARNA: Interactive drawing and editing of the RNA secondary structure. Bioinformatics, 25(15), 1974–1975. 10.1093/bioinformatics/btp250 19398448 PMC2712331

[ece39155-bib-0025] de Vargas, C. , Audic, S. , Henry, N. , Decelle, J. , Mahé, F. , Logares, R. , Lara, E. , Berney, C. , le Bescot, N. , Probert, I. , Carmichael, M. , Poulain, J. , Romac, S. , Colin, S. , Aury, J. M. , Bittner, L. , Chaffron, S. , Dunthorn, M. , Engelen, S. , … Karsenti, E. (2015). Eukaryotic plankton diversity in the sunlit ocean. Science, 348(6237). 10.1126/science.1261605 25999516

[ece39155-bib-0026] de Wit, R. , & Bouvier, T. (2006). 'Everything is everywhere, but, the environment selects'; what did Baas Becking and Beijerinck really say? Environmental Microbiology, 8, 755–758. 10.1111/j.1462-2920.2006.01017.x 16584487

[ece39155-bib-0027] Devaux, C. , & Lande, R. (2008). Incipient allochronic speciation due to non‐selective assortative mating by flowering time, mutation and genetic drift. Proceedings of the Royal Society of London, Series B: Biological Sciences, 275(1652), 2723–2732. 10.1098/rspb.2008.0882 PMC2605824 18700202

[ece39155-bib-0028] Di Dato, V. , Musacchia, F. , Petrosino, G. , Patil, S. , Montresor, M. , Sanges, R. , & Ferrante, M. I. (2015). Transcriptome sequencing of three *Pseudo‐nitzschia* species reveals comparable gene sets and the presence of nitric oxide synthase genes in diatoms. Scientific Reports. 10.1038/srep12329 PMC4648414 26189990

[ece39155-bib-0029] Doledec, S. , Chessel, D. , & Gimaret‐Carpentier, C. (2000). Niche separation in community analysis: A new method. Ecology, 81(10), 2914–2927. 10.1890/0012-9658(2000)081[2914:NSICAA]2.0.CO;2

[ece39155-bib-0030] Dong, H. C. , Lundholm, N. , Teng, S. T. , Li, A. , Wang, C. , Hu, Y. , & Li, Y. (2020). Occurrence of *Pseudo‐nitzschia* species and associated domoic acid production along the Guangdong coast, South China Sea. Harmful Algae, 98, 101899. 33129456 10.1016/j.hal.2020.101899

[ece39155-bib-0031] Edler, L. , & Elbrächter, M. (2010). The Utermöhl method for quantitative phytoplankton analysis. In B. Karlson , C. K. Cusack , & E. Bresnan (Eds.), Microscopic and molecular methods for quantitative phytoplankton analysis (Vol. 55, pp. 13–20). UNESCO.

[ece39155-bib-0032] Fenchel, T. (2005). Cosmopolitan microbes and their ‘cryptic’ species. Aquatic Microbial Ecology, 41, 49–54.

[ece39155-bib-0033] Fenchel, T. , & Finlay, B. J. (2004). The ubiquity of small species: Patterns of local and global diversity. Bioscience, 54(8), 777–784. 10.1641/0006-3568(2004)054[0777:tuossp]2.0.co;2

[ece39155-bib-0034] Forrest, J. , & Miller‐Rushing, A. J. (2010). Toward a synthetic understanding of the role of phenology in ecology and evolution. Philosophical Transaction of The Royal Society, 365, 3101–3112. 10.1098/rstb.2010.0145 PMC2981948 20819806

[ece39155-bib-0035] Fortunato, A. E. , Annunziata, R. , Jaubert, M. , Bouly, J.‐P. , & Falciatore, A. (2015). Dealing with light: The widespread and multitasking cryptochrome/photolyase family in photosynthetic organisms. Journal of Plant Physiology, 172, 42–54. 10.1016/j.jplph.2014.06.011 25087009

[ece39155-bib-0036] Foulon, E. , Not, F. , Jalabert, F. , Cariou, T. , Massana, R. , & Simon, N. (2008). Ecological niche partitioning in the picoplanktonic green alga *Micromonas pusilla*: Evidence from environmental surveys using phylogenetic probes. Environmental Microbiology, 10(9), 2433–2443. 18537812 10.1111/j.1462-2920.2008.01673.x

[ece39155-bib-0037] Gai, F. , Hedemand, C. , Louw, D. , Grobler, K. , Krock, B. , Moestrup, Ø. , & Lundholm, N. (2018). Morphological, molecular and toxigenic characteristics of Namibian *Pseudo‐nitzschia* species ‐ including *Pseudo‐nitzschia bucculenta* sp. nov. Harmful Algae, 76, 80–95. 29887207 10.1016/j.hal.2018.05.003

[ece39155-bib-0038] Gaonkar, C. C. , Piredda, R. , Sarno, D. , Zingone, A. , Montresor, M. , & Kooistra, W. H. C. F. (2020). Species detection and delineation in the marine planktonic diatoms *Chaetoceros* and *Bacteriastrum* through metabarcoding: Making biological sense of haplotype diversity. Environmental Microbiology, 22(5), 1917–1929. 10.1111/1462-2920.14984 32157787

[ece39155-bib-0039] Giner, C. R. , Balagué, V. , Krabberød, A. K. , Ferrera, I. , Reñé, A. , Garcés, E. , Gasol, J. M. , Logares, R. , & Massana, R. (2019). Quantifying long‐term recurrence in planktonic microbial eukaryotes. Molecular Ecology, 28, 923–935. 10.1111/mec.14929 30411822

[ece39155-bib-0040] Giulietti, S. , Romagnoli, T. , Siracusa, M. , Bacchiocchi, S. , Totti, C. , & Accoroni, S. (2021). Integrative taxonomy of the *P* *seudo‐nitzschia* (Bacillariophyceae) populations in the NW Adriatic Sea, with a focus on a novel cryptic species in the *P. delicatissima* species complex. Phycologia, 1–18. 10.1080/00318884.2021.1899733

[ece39155-bib-0041] Guiry, M. D. , & Guiry, G. M. (2022). AlgaeBase. World‐wide electronic publication, National University of Ireland. https://www.algaebase.org

[ece39155-bib-0042] Hendry, A. P. (2009). Speciation. Nature, 458(7235), 162–164. 10.1038/458162a 19279629

[ece39155-bib-0043] Hendry, A. P. , & Day, T. (2005). Population structure attributable to reproductive time: Isolation by time and adaptation by time. Molecular Ecology, 14, 901–916. 10.1111/j.1365-294X.2005.02480.x 15773924

[ece39155-bib-0044] Hendry, A. P. , Nosil, P. , & Rieseberg, L. H. (2007). The speed of ecological speciation. Functional Ecology, 21(3), 455–464. 19096732 10.1111/j.1365-2435.2006.01240.x PMC2605086

[ece39155-bib-0045] Huang, C. X. , Dong, H. C. , Lundholm, N. , Teng, S. T. , Zheng, G. C. , Tan, Z. J. , Lim, P. T. , & Li, Y. (2019). Species composition and toxicity of the genus *Pseudo‐nitzschia* in Taiwan Strait, including *P. chiniana* sp. nov. and *P. qiana* sp. nov. Harmful Algae, 84, 195–209. 31128805 10.1016/j.hal.2019.04.003

[ece39155-bib-0046] Hutchinson, G. E. (1961). The paradox of the plankton. The American Naturalist, XCV(882), 137–145.

[ece39155-bib-0047] Katoh, K. , & Standley, D. M. (2013). MAFFT multiple sequence alignment software version 7: Improvements in performance and usability. Molecular Biology and Evolution, 30(4), 772–780. 10.1093/molbev/mst010 23329690 PMC3603318

[ece39155-bib-0048] Kumar, S. , Stecher, G. , Li, M. , Knyaz, C. , & Tamura, K. (2018). MEGA X: Molecular evolutionary genetics analysis across computing platforms. Molecular Biology and Evolution, 35(6), 1547–1549. 10.1093/molbev/msy096 29722887 PMC5967553

[ece39155-bib-0049] Lamari, N. , Ruggiero, M. V. , d'Ippolito, G. , Kooistra, W. H. C. F. , Fontana, A. , & Montresor, M. (2013). Specificity of lipoxygenase pathways supports species delineation in the marine diatom genus *Pseudo‐nitzschia* . PLoS One, 8(8), 73281. 10.1371/journal.pone.0073281 PMC3754938 24014077

[ece39155-bib-0050] Lambert, S. , Tragin, M. , Lozano, J.‐C. , Ghiglione, J.‐F. , Vaulot, D. , Bouget, F.‐Y. , & Galand, P. E. (2019). Rhythmicity of coastal marine picoeukaryotes, bacteria and archaea despite irregular environmental perturbations. The ISME Journal, 13(2), 388. 10.1038/s41396-018-0281-z 30254323 PMC6331585

[ece39155-bib-0051] Lebret, K. , Kritzberg, E. S. , Figueroa, R. , & Rengefors, K. (2012). Genetic diversity within and genetic differentiation between blooms of a microalgal species. Environmental Microbiology, 14(9), 2395–2404. 10.1111/j.1462-2920.2012.02769.x 22568551 PMC3466416

[ece39155-bib-0052] Letunic, I. , & Bork, P. (2019). Interactive tree of life (iTOL) v4: Recent updates and new developments. Nucleic Acids Research, 47(W1), 256–259. 10.1093/nar/gkz239 PMC6602468 30931475

[ece39155-bib-0053] Lim, H.‐C. , Leaw, C.‐P. , Su, S. N.‐P. , Teng, S.‐T. , Usup, G. , Mohammad‐Noor, N. , Lundholm, N. , Kotaki, Y. , & Lim, P. T. (2012). Morphology and molecular characterization of *Pseudo‐nitzschia* (Bacillariophyceae) from Malaysian Borneo, including the new species *Pseudo‐nitzschia circumpora* sp. nov. Journal of Phycology, 48, 1232–1247. 10.1111/j.1529-8817.2012.01213.x 27011282

[ece39155-bib-0054] Lim, H. C. , Tan, S. N. , Teng, S. T. , Lundholm, N. , Orive, E. , David, H. , Quijano‐Scheggia, S. , Leong, S. C. Y. , Wolf, M. , Bates, S. S. , Lim, P. T. , & Leaw, C. P. (2018). Phylogeny and species delineation in the marine diatom *Pseudo‐nitzschia* (Bacillariophyta) using cox1, LSU and ITS2 rRNA genes: A perspective in character evolution. Journal of Phycology, 54, 234–248. 10.1111/jpy.12620 29377161

[ece39155-bib-0055] Limardo, A. J. , Sudek, S. , Choi, C. J. , Poirier, C. , Rii, Y. M. , Blum, M. , Roth, R. , Goodenough, U. , Church, M. J. , & Worden, A. Z. (2017). Quantitative biogeography of picoprasinophytes establishes ecotype distributions and significant contributions to marine phytoplankton. Environmental Microbiology, 19(8), 3219–3234. 10.1111/1462-2920.13812 28585420

[ece39155-bib-0056] Longobardi, L. , Dubroca, L. , Margiotta, F. , Sarno, D. , & Zingone, A. (2022). Photoperiod‐driven rhythms reveal multi‐decadal stability of phytoplankton communities in a highly fluctuating coastal environment. Scientific Reports, 12, 3908. 10.1038/s41598-022-07009-6 35273208 PMC8913669

[ece39155-bib-0057] Lundholm, N. , Moestrup, Ø. , Kotaki, Y. , Hoef‐Emden, K. , Scholin, C. , & Miller, P. (2006). Inter‐ and intraspecific variation of the *Pseudo‐nitzschia delicatissima* complex (Bacillariophyceae) illustrated by rRNA probes, morphological data and phylogenetic analyses. Journal of Phycology, 42(2), 464–481. 10.1111/j.1529-8817.2006.00211.x

[ece39155-bib-0058] Mai, J. C. , & Coleman, A. W. (1997). The internal transcribed spacer 2 exhibits a common secondary structure in green algae and flowering plants. Journal of Molecular Evolution, 44(3), 258–271. 9060392 10.1007/pl00006143

[ece39155-bib-0059] McDonald, S. M. (2007). Temporal molecular diversity of marine phytoplankton. (PhD Thesis). The Open University.

[ece39155-bib-0060] McDonald, S. M. , Sarno, D. , & Zingone, A. (2007). Identifying *Pseudo‐nitzschia* species in natural samples using genus‐specific PCR primers and clone libraries. Harmful Algae, 6, 849–860. 10.1016/j.hal.2007.03.003

[ece39155-bib-0061] Medlin, L. K. , Elwood, H. J. , Stickel, S. , & Sogin, M. L. (1988). The characterization of enzymatically amplified eukaryotic 16S‐like rRNA‐coding regions. Gene, 71, 491–499. 3224833 10.1016/0378-1119(88)90066-2

[ece39155-bib-0062] Montresor, M. , Vitale, L. , D'Alelio, D. , & Ferrante, M. I. (2016). Sex in marine planktonic diatoms: Insights and challenges. Perspectives in Phycology, 3, 61–75. 10.1127/pip/2016/0045

[ece39155-bib-0063] Moon‐van der Staay, S. Y. , De Wachter, R. , & Vaulot, D. (2001). Oceanic 18S rDNA sequences from picoplankton reveal unsuspected eukaryotic diversity. Nature, 409, 607–610. 11214317 10.1038/35054541

[ece39155-bib-0064] Nanjappa, D. , Kooistra, W. H. C. F. , & Zingone, A. (2013). A reappraisal of the genus *Leptocylindrus* (Bacillariophyta), with the addition of three species and the erection of *Tenuicylindrus* gen. Nov. Journal of Phycology, 49, 917–936. 10.1111/jpy.12102 27007316

[ece39155-bib-0065] Orsini, L. , Procaccini, G. , Sarno, D. , & Montresor, M. (2004). Multiple rDNA ITS‐types within the diatom *Pseudo‐nitzschia delicatissima* (Bacillariophyceae) and their relative abundances across a spring bloom in the Gulf of Naples. Marine Ecology Progress Series, 271, 87–98.

[ece39155-bib-0066] Orsini, L. , Sarno, D. , Procaccini, G. , Poletti, R. , Dahlmann, J. , & Montresor, M. (2002). Toxic *Pseudo‐nitzschia multistriata* (Bacillariophyceae) from the Gulf of Naples: Morphology, toxin analysis and phylogenetic relationships with other *Pseudo‐nitzschia* species. European Journal of Phycology, 37, 247–257.

[ece39155-bib-0067] Orsini, L. , Vanoverbeke, J. , Swillen, I. , Mergeay, J. , & De Meester, L. (2013). Drivers of population genetic differentiation in the wild: Isolation by dispersal limitation, isolation by adaptation and isolation by colonization. Molecular Ecology, 22(24), 5983–5999. 24128305 10.1111/mec.12561

[ece39155-bib-0068] Palenik, B. , Grimwood, J. , Aerts, A. , Rouzé, P. , Salamov, A. , Putnam, N. , Dupont, C. , Jorgensen, R. , Derelle, E. , Rombauts, S. , Zhou, K. , Otillar, R. , Merchant, S. S. , Podell, S. , Gaasterland, T. , Napoli, C. , Gendler, K. , Manuell, A. , Tai, V. , … Grigoriev, I. V. (2007). The tiny eukaryote *Ostreococcus* provides genomic insights into the paradox of plankton speciation. Proceedings of the National Academy of Sciences, 104(18), 7705–7710. 10.1073/pnas.0611046104 PMC1863510 17460045

[ece39155-bib-0069] Palumbi, S. R. (1994). Genetic divergence, reproductive isolation, and marine speciation. Annual Review of Ecology and Systematics, 25(1), 547–572.

[ece39155-bib-0070] Pargana, A. , Musacchia, F. , Sanges, R. , Russo, M. T. , Ferrante, M. I. , Bowler, C. , & Zingone, A. (2020). Intraspecific diversity in the cold stress response of transposable elements in the diatom *Leptocylindrus aporus* . Genes, 11, 9. 10.3390/genes11010009 PMC7017206 31861932

[ece39155-bib-0071] Piganeau, G. , Eyre‐Walker, A. , Grimsley, N. , & Moreau, H. (2011). How and why DNA barcodes underestimate the diversity of microbial eukaryotes. PLoS One, 6(2), e16342. 21347361 10.1371/journal.pone.0016342 PMC3037371

[ece39155-bib-0072] Potkamp, G. , & Fransen, C. (2019). Speciation with gene flow in marine systems. Contributions to Zoology, 1–40. 10.1163/18759866-20191344

[ece39155-bib-0073] Priisholm, K. , Moestrup, Ø. , & Lundholm, N. (2002). Taxonomic notes on the marine diatom genus *Pseudo‐nitzschia* in the Andaman Sea near the Island of Phuket, Thailand, with a description of *Pseudo‐nitzschia micropora* sp. nov. Diatom Research, 17, 153–175.

[ece39155-bib-0074] Pugliese, L. , Casabianca, S. , Perini, F. , Andreoni, F. , & Penna, A. (2017). A high resolution melting method for the molecular identification of the potentially toxic diatom *Pseudo‐nitzschia* spp. in the Mediterranean Sea. Scientific Reports, 7(1), 1–10. 10.1038/s41598-017-04245-z 28652566 PMC5484702

[ece39155-bib-0075] Quijano‐Scheggia, S. I. , Garcés, E. , Lundholm, N. , Moestrup, O. , Andree, K. , & Camp, J. (2009). Morphology, physiology, molecular phylogeny and sexual compatibility of the cryptic *Pseudo‐nitzschia delicatissima* complex (Bacillariophyta), including the description of *P. arenysensis* sp. nov. Phycologia, 48(6), 492–509. 10.2216/08-21.1

[ece39155-bib-0076] Rengefors, K. , Kremp, A. , Reusch, T. B. H. , & Wood, M. (2017). Genetic diversity and evolution in eukaryotic phytoplankton: Revelations from population genetic studies. Journal of Plankton Research. 10.1093/plankt/fbw098

[ece39155-bib-0077] Reuter, J. S. , & Mathews, D. H. (2010). RNAstructure: Software for RNA secondary structure prediction and analysis. BMC Bioinformatics, 11(1), 129. 10.1186/1471-2105-11-129 20230624 PMC2984261

[ece39155-bib-0078] Ribera d'Alcalà, M. , Conversano, F. , Corato, F. , Licandro, P. , Mangoni, O. , Marino, D. , Mazzocchi, M. G. , Modigh, M. , Montresor, M. , Nardella, M. , & Saggiomo, V. (2004). Seasonal patterns in plankton communities in a pluriannual time series at a coastal Mediterranean site (gulf of Naples): An attempt to discern recurrences and trends. Scientia Marina, 68(Suppl. 1), 65–83.

[ece39155-bib-0079] Richlen, M. L. , Erdner, D. L. , McCauley, L. A. R. , Libera, K. , & Anderson, D. M. (2012). Extensive genetic diversity and rapid population differentiation during blooms of *Alexandrium fundyense* (Dinophyceae) in an isolated salt pond on Cape Cod, MA, USA. Ecology and Evolution, 2(10), 2583–2594. 10.1002/ece3.373 PMC3492784 23145343

[ece39155-bib-0080] Rosser, N. L. (2016). Demographic history and asynchronous spawning shape genetic differentiation among populations of the hard coral *Acropora tenuis* in Western Australia. Molecular Phylogenetics and Evolution, 98, 89–96. 10.1016/j.ympev.2016.02.004 26876640

[ece39155-bib-0081] Round, F. E. , Crawford, R. M. , & Mann, D. G. (1990). The diatoms. Biology and morphology of the genera. Cambridge University Press.

[ece39155-bib-0082] Ruggiero, M. , Kooistra, W. , Piredda, R. , Sarno, D. , Zampicinini, G. , Zingone, A. , & Montresor, M. (2022). Temporal changes of genetic structure and diversity in a marine diatom genus discovered via metabarcoding. Environmental DNA, 1–13. 10.1002/edn3.288

[ece39155-bib-0083] Ruggiero, M. V. , D'Alelio, D. , Ferrante, M. I. , Santoro, M. , Vitale, L. , Procaccini, G. , & Montresor, M. (2018). Clonal expansion behind a marine diatom bloom. The ISME Journal, 12(2), 463–472. 10.1038/ismej.2017.181 29160864 PMC5776461

[ece39155-bib-0084] Ruggiero, M. V. , Sarno, D. , Barra, L. , Kooistra, W. H. C. F. , Montresor, M. , & Zingone, A. (2015). Diversity and temporal pattern of *Pseudo‐nitzschia* species (Bacillariophyceae) through the molecular lens. Harmful Algae, 42(0), 15–24. 10.1016/j.hal.2014.12.001

[ece39155-bib-0085] Rynearson, T. , Newton, J. , & Armbrust, E. (2006). Spring bloom development, genetic variation, and population succession in the planktonic diatom *Ditylum brightwellii* . Limnology and Oceanography, 51(3), 1249–1261.

[ece39155-bib-0086] Sabia, L. , Costanzo, A. , Ribera d'Alcalà, M. , Saggiomo, V. , Zingone, A. , & Margiotta, F. (2019). Assessing the quality of biogeochemical coastal data: A step‐wise procedure. Mediterranean Marine Science, 20(1), 56–73. 10.12681/mms.15935

[ece39155-bib-0087] Sarno, D. , Kooistra, W. C. H. F. , Balzano, S. , Hargraves, P. E. , & Zingone, A. (2007). Diversity in the genus *Skeletonema* (Bacillariophyceae): III. Phylogenetic position and morphological variability of *Skeletonema costatum* and *Skeletonema grevillei*, with the description of *Skeletonema ardens* sp. nov. Journal of Phycology, 43, 156–170. 10.1111/j.1529-8817.2006.00305.x

[ece39155-bib-0088] Sarno, D. , Kooistra, W. C. H. F. , Medlin, L. K. , Percopo, I. , & Zingone, A. (2005). Diversity in the genus *Skeletonema* (Bacillariophyceae). II. An assessment of the taxonomy of *S. costatum*‐like species, with the description of four new species. Journal of Phycology, 41, 151–176. 10.1111/j.1529-8817.2005.04067.x

[ece39155-bib-0089] Sarno, D. , Zingone, A. , & Montresor, M. (2010). A massive and simultaneous sex event of two *Pseudo‐nitzschia* species. Deep Sea Research (Part II, Topical Studies in Oceanography), 57, 248–255. 10.1016/j.dsr2.2009.09

[ece39155-bib-0090] Sassenhagen, I. , Gao, Y. , Lozano‐Duque, Y. , Parsons, M. L. , Smith, T. B. , & Erdner, D. L. (2018). Comparison of spatial and temporal genetic differentiation in a harmful dinoflagellate species emphasizes impact of local processes. Frontiers in Marine Science, 5, 393. 10.3389/fmars.2018.00393

[ece39155-bib-0091] Schaum, C.‐E. , Buckling, A. , Smirnoff, N. , Studholme, D. , & Yvon‐Durocher, G. (2018). Environmental fluctuations accelerate molecular evolution of thermal tolerance in a marine diatom. Nature Communications, 9(1), 1–14. 10.1038/s41467-018-03906-5 PMC5928086 29712900

[ece39155-bib-0092] Seibel, P. N. , Müller, T. , Dandekar, T. , & Wolf, M. (2008). Synchronous visual analysis and editing of RNA sequence and secondary structure alignments using 4SALE. BMC Research Notes, 1(1), 1–7. 18854023 10.1186/1756-0500-1-91 PMC2587473

[ece39155-bib-0093] Simon, N. , Foulon, E. , Grulois, D. , Six, C. , Desdevises, Y. , Latimier, M. , le Gall, F. , Tragin, M. , Houdan, A. , Derelle, E. , Jouenne, F. , Marie, D. , le Panse, S. , Vaulot, D. , & Marin, B. (2017). Revision of the genus *Micromonas* Manton et Parke (Chlorophyta, Mamiellophyceae), of the type species *M. pusilla* (Butcher) Manton & Parke and of the species *M. commoda* van Baren, Bachy and Worden and description of two new species based on the genetic and phenotypic characterization of cultured isolates. Protist, 168(5), 612–635. 10.1016/j.protis.2017.09.002 29028580

[ece39155-bib-0094] Sjoqvist, C. , Godhe, A. , Jonsson, P. R. , Sundqvist, L. , & Kremp, A. (2015). Local adaptation and oceanographic connectivity patterns explain genetic differentiation of a marine diatom across the North Sea‐Baltic Sea salinity gradient. Molecular Ecology, 24(11), 2871–2885. 10.1111/mec.13208 25892181 PMC4692096

[ece39155-bib-0095] Tammilehto, A. , Watts, C. P. , & Lundholm, N. (2016). Isolation by time during an arctic phytoplankton spring bloom. Journal of Eukaryotic Microbiology, 64(2), 248–256. 10.1111/jeu.12356 27543207

[ece39155-bib-0096] Teng, S. T. , Lim, P. T. , Lim, H. C. , Rivera‐Vilarelle, M. , Quijano‐Scheggia, S. , Takata, Y. , Quilliam, M. A. , Wolf, M. , Bates, S. S. , & Leaw, C. P. (2015). A non‐toxigenic but morphologically and phylogenetically distinct new species of *Pseudo‐nitzschia*, *P. sabit* sp. nov. (Bacillariophyceae). Journal of Phycology, 51(4), 706–725. 10.1111/jpy.12313 26986792

[ece39155-bib-0097] Tesson, S. V. M. , Borra, M. , Kooistra, W. , & Procaccini, G. (2011). Microsatellite primers in the planktonic diatom *Pseudo‐nitzschia multistriata* (Bacillariophyceae). American Journal of Botany, 98(2), 33–35. 10.3732/ajb.1000430 21613102

[ece39155-bib-0098] Tesson, S. V. M. , Montresor, M. , Procaccini, G. , & Kooistra, W. H. C. F. (2014). Temporal changes in population structure of a marine planktonic diatom. PLoS One. 10.1371/journal.pone.0114984 PMC4266644 25506926

[ece39155-bib-0099] Weis, A. E. , & Kossler, T. M. (2004). Genetic variation in flowering time induces phenological assortative mating: Quantitative genetic methods applied to *Brassica rapa* . American Journal of Botany, 91(6), 825–836. 21653438 10.3732/ajb.91.6.825

[ece39155-bib-0100] White, T. J. , Bruns, T. , Lee, S. , & Taylor, J. (1990). Amplification and direct sequencing of fungal ribosomal RNA genes for phylogenetics. In M. A. Innis , D. H. Gelfand , J. J. Sninsky , & T. J. White (Eds.), PCR protocols (pp. 315–322). Academic Press.

[ece39155-bib-0101] Whittaker, K. A. , & Rynearson, T. A. (2017). Evidence for environmental and ecological selection in a microbe with no geographic limits to gene flow. Proceedings of the National Academy of Sciences of the United States of America, 114(10), 2651–2656. 10.1073/pnas.1612346114 28209775 PMC5347567

[ece39155-bib-0102] Zenetos, A. , Gofas, S. , Verlaque, M. , Çinar, M. E. , García Raso, J. E. , Bianchi, C. N. , Morri, C. , Azzurro, E. , Bilecenoglu, M. , Froglia, C. , & Siokou‐Frangou, I. (2010). Alien species in the Mediterranean Sea by 2010. A contribution to the application of European Union's marine strategy framework directive (MSFD). Part I. spatial distribution. Mediterranean Marine Science, 11(2), 381–493. 10.12681/mms.87

[ece39155-bib-0103] Zingone, A. , D'Alelio, D. , Mazzocchi, M. G. , Montresor, M. , Sarno, D. , & LTER‐MC Team . (2019). Time series and beyond: Multifaceted plankton research at a marine Mediterranean LTER site. Nature Conservation, 34, 273. 10.3897/natureconservation.34.30789

[ece39155-bib-0104] Zingone, A. , Siano, R. , D'Alelio, D. , & Sarno, D. (2006). Potentially toxic and harmful microalgae from coastal waters of the Campania region (Tyrrhenian Sea, Mediterranean Sea). Harmful Algae, 5, 321–337. 10.1016/j.hal.2005.09.002

