# Supplementary material for: Phenological segregation suggests speciation by time in the planktonic diatom Pseudo‐nitzschia allochrona sp. nov
Source: Ecol Evol. 2022 Aug 4;12(8):e9155. doi: 10.1002/ece3.9155 (PMC9352866; doi:10.1002/ece3.9155)
Supplement: Supplementary file 1 — Table S1 [file ECE3-12-e9155-s005.docx]

Table A1: List of strains of *Pseudo-nitzschia allochrona* used in the present study for electron microscopy (EM), domoic acid (DA) analyses and mating experiments (Mating). All strains were characterized through at least one of the following molecular markers: 28S, ITS, 18S and *rbc*L, as indicated in the table.

| Strain name | Site | Station Coordinates  Latitude [°N]-Longitude [°E] | Isolation date | EM | DA | Mating | 28S | ITS | 18S | rbcL |
| --- | --- | --- | --- | --- | --- | --- | --- | --- | --- | --- |
| 1. SZN-B367 | LTER-MC | 40º 48.50’-14º 15.00’ | 31/7/ 2007 |  |  |  | X |  |  |  |
| 1. SZN-B366 | LTER-MC | 40º 48.50’-14º 15.00’ | 31/7/ 2007 |  |  |  | X |  |  |  |
| 1. SZN-B365 | LTER-MC | 40º 48.50’-14º 15.00’ | 31/7/ 2007 |  |  |  | X |  |  |  |
| 1. SZN-B364 | LTER-MC | 40º 48.50’-14º 15.00’ | 31/7/ 2007 |  |  |  | X |  |  |  |
| 1. SZN-B359 | LTER-MC | 40º 48.50’-14º 15.00’ | 28/08/2007 |  |  |  | X |  |  |  |
| 1. SZN-B363 | LTER-MC | 40º 48.50’-14º 15.00’ | 28/08/2007 |  |  |  | X |  |  |  |
| 1. SZN-B361 | LTER-MC | 40º 48.50’-14º 15.00’ | 28/08/2007 |  |  |  | X |  |  |  |
| 1. SZN-B358 | LTER-MC | 40º 48.50’-14º 15.00‘ | 28/08/2007 |  |  |  | X |  |  |  |
| 1. SZN-B362 | LTER-MC | 40º 48.50’-14º 15.00’ | 28/08/2007 |  |  |  | X |  |  |  |
| 1. MC784_4II_2 | LTER-MC | 40º 48.50’-14º 15.00‘ | 02/10/2007 |  |  |  | X |  |  |  |
| 1. MC784_4II | LTER-MC | 40º 48.50’-14º 15.00‘ | 02/10/2007 |  |  |  | X |  |  |  |
| 1. MC784 B6 | LTER-MC | 40º 48.50’-14º 15.00‘ | 02/10/2007 |  |  |  | X |  |  |  |
| 1. MC784 B2 | LTER-MC | 40º 48.50’-14º 15.00‘ | 02/10/2007 |  |  |  | X |  |  |  |
| 1. MC784 A3 | LTER-MC | 40º 48.50’-14º 15.00’ | 02/10/2007 |  |  |  | X |  |  |  |
| 1. SZN-B495 | Ionian Sea | 39º 46.00’-19º 07.06’ | 21/09/2008 | X | X |  | X |  |  | X |
| 1. SZN-B853 | Ionian Sea | 39°46.00’-19°07.06’ | 21/09/2008 | X |  |  | X | X |  | X |
| 1. 21ott08strain3 | LTER-MC | 40º 48.50’-14º 15.00’ | 21/10/2008 |  |  |  | X |  |  |  |
| 1. SZN-B497 | LTER-MC | 40º 48.50’-14º 15.00’ | 28/07/2009 | X |  |  | X |  |  |  |
| 1. SZN-B498 | LTER-MC | 40º 48.50’-14º 15.00’ | 28/07/2009 | X |  |  | X |  |  |  |
| 1. SZN-B499 | LTER-MC | 40º 48.50’-14º 15.00’ | 28/07/2009 | X |  |  | X | X |  | X |
| 1. SZN-B500 | LTER-MC | 40º 48.50’-14º 15.00’ | 28/07/2009 | X |  |  | X |  |  |  |
| 1. SZN-B501 | LTER-MC | 40º 48.50’-14º 15.00’ | 28/07/2009 | X |  |  | X | X |  | X |
| 1. SZN-B503 | LTER-MC | 40º 48.50’-14º 15.00’ | 28/07/2009 | X |  |  | X |  |  |  |
| 1. SZN-B504 | LTER-MC | 40º 48.50’-14º 15.00’ | 28/07/2009 | X |  |  | X |  |  |  |
| 1. SZN-B507 | LTER-MC | 40º 48.50’-14º 15.00’ | 28/07/2009 | X |  |  | X |  |  |  |
| 1. SZN-B509 | LTER-MC | 40º 48.50’-14º 15.00’ | 28/07/2009 | X |  |  | X | X |  | X |
| 1. SZN-B514 | LTER-MC | 40º 48.50’-14º 15.00’ | 04/08/2009 | X |  |  | X |  |  |  |
| 1. SZN-B522 | LTER-MC | 40º 48.50’-14º 15.00’ | 29/09/2009 |  |  |  | X |  |  |  |
| 1. SZN-B523 | LTER-MC | 40º 48.50’-14º 15.00’ | 29/09/2009 | X |  |  | X |  |  |  |
| 1. SZN-B524 | LTER-MC | 40º 48.50’-14º 15.00’ | 29/09/2009 |  | X |  | X | X |  | X |
| 1. SZN-B536 | LTER-MC | 40º 48.50’-14º 15.00’ | 29/09/2009 |  |  |  | X |  |  |  |
| 1. SZN-B535 | LTER-MC | 40º 48.50’-14º 15.00’ | 29/09/2009 |  |  |  | X |  |  |  |
| 1. SZN-B534 | LTER-MC | 40º 48.50’-14º 15.00’ | 29/09/2009 |  |  |  | X |  |  |  |
| 1. SZN-B521 | LTER-MC | 40º 48.50’-14º 15.00’ | 29/09/2009 |  |  |  | X |  |  |  |
| 1. SZN-B525 | LTER-MC | 40º 48.50’-14º 15.00’ | 06/10/2009 |  |  |  | X | X |  | X |
| 1. SZN-B526 | LTER-MC | 40º 48.50’-14º 15.00’ | 06/10/2009 |  |  |  | X |  |  |  |
| 1. SZN-B527 | LTER-MC | 40º 48.50’-14º 15.00’ | 06/10/2009 |  |  |  | X |  |  |  |
| 1. SZN-B537 | LTER-MC | 40º 48.50’-14º 15.00’ | 06/10/2009 |  |  |  | X |  |  |  |
| 1. SZN-B540 | LTER-MC | 40º 48.50’-14º 15.00’ | 04/11/2009 |  |  |  | X |  |  |  |
| 1. SZN-B541 | LTER-MC | 40º 48.50’-14º 15.00’ | 17/11/2009 |  |  |  | X |  |  |  |
| 1. SZN-B631 | Gulf of Naples | 40º 48.50’-14º 15.00’ | --//11/2010 |  |  |  |  |  | X |  |
| 1. Int-3I-A3 | Mergellina harbour, Gulf of Naples | 40º 49.53’-13º 86.00’ | 05/10/2015 |  |  |  |  | X |  |  |
| 1. Int-3I-A4 | Mergellina harbour, Gulf of Naples | 40º 49.53’-13º 86.00’ | 05/10/2015 |  |  |  |  | X |  |  |
| 1. MC1177-D5 | LTER-MC | 40º 48.50’-14º 15.00’ | 10/10/2015 |  |  |  |  | X |  |  |
| 1. MC1177-D6 | LTER-MC | 40º 48.50’-14º 15.00’ | 10/10/2015 |  |  |  |  | X |  |  |
| 1. MC1177-C6 | LTER-MC | 40º 48.50’-14º 15.00’ | 10/10/2015 |  |  |  |  | X |  |  |
| 1. MC1028-B5 | LTER-MC | 40º 48.50’-14º 15.00’ | 05/07/2016 |  |  | X |  | X |  |  |
| 1. MC1028-C3 | LTER-MC | 40º 48.50’-14º 15.00’ | 05/07/2016 |  |  | X |  | X |  |  |
| 1. MC1028-C5 | LTER-MC | 40º 48.50’-14º 15.00’ | 05/07/2016 |  |  | X |  | X |  |  |
| 1. MC1209-A1 | LTER-MC | 40º 48.50’-14º 15.00’ | 12/07/2016 |  |  | X |  | X |  |  |
| 1. MC1209-A2 | LTER-MC | 40º 48.50’-14º 15.00’ | 12/07/2016 |  |  | X |  | X |  |  |
| 1. MC1209-A4 | LTER-MC | 40º 48.50’-14º 15.00’ | 12/07/2016 |  |  | X |  | X |  |  |
| 1. MC1209-B1 | LTER-MC | 40º 48.50’-14º 15.00’ | 12/07/2016 |  |  | X |  | X |  |  |
| 1. MC1209-B3 | LTER-MC | 40º 48.50’-14º 15.00’ | 12/07/2016 |  |  | X |  | X |  |  |
| 1. MC1209-B4 | LTER-MC | 40º 48.50’-14º 15.00’ | 12/07/2016 |  |  | X |  | X |  |  |
| 1. MC1209-B6 | LTER-MC | 40º 48.50’-14º 15.00’ | 12/07/2016 |  |  | X |  | X |  |  |
| 1. MC1209-C1 | LTER-MC | 40º 48.50’-14º 15.00’ | 12/07/2016 |  |  | X |  | X |  |  |
| 1. MC1209-C2 | LTER-MC | 40º 48.50’-14º 15.00’ | 12/07/2016 |  |  | X |  | X |  |  |
| 1. MC1209-C3 | LTER-MC | 40º 48.50’-14º 15.00’ | 12/07/2016 |  |  | X |  | X |  |  |
| 1. MC1209-C3/A | LTER-MC | 40º 48.50’-14º 15.00’ | 12/07/2016 |  |  | X |  | X |  |  |
| 1. MC1209-C4 | LTER-MC | 40º 48.50’-14º 15.00’ | 12/07/2016 |  |  | X |  | X |  |  |
| 1. MC1209-C5 | LTER-MC | 40º 48.50’-14º 15.00’ | 12/07/2016 |  |  | X |  | X |  |  |
| 1. MC1209-D3 | LTER-MC | 40º 48.50’-14º 15.00’ | 12/07/2016 |  |  | X |  | X |  |  |
| 1. MC1209-D4 | LTER-MC | 40º 48.50’-14º 15.00’ | 12/07/2016 |  |  | X |  | X |  |  |
