# Supplementary material for: Phenological segregation suggests speciation by time in the planktonic diatom Pseudo‐nitzschia allochrona sp. nov
Source: Ecol Evol. 2022 Aug 4;12(8):e9155. doi: 10.1002/ece3.9155 (PMC9352866; doi:10.1002/ece3.9155)
Supplement: Supplementary file 2 — Table S2 [file ECE3-12-e9155-s002.docx]

Table A2: GenBank accession numbers of the strains used for phylogenetic analyses for each molecular marker.

| **18 S** | | |
| --- | --- | --- |
| **Species** | **Strain designation** | **GB Accession Number** |
| *Fragilariopsis curta* | Strain 3 | EF140623 |
| *Fragilariopsis cylindrus* | NIES-3887 | LC189151 |
| *P. allochrona* | SZN-B631 | KJ608076 |
| *P. americana* | SKLMP_Sh004 | MG799146 |
| *P. arctica* | RCC2004 | JF794046 |
| *P. arenysensis* | SZN-B593 | OK135731 |
| *P. australis* | SPC21 | GU373961 |
| *P. australis* | POMXaus | AM235384 |
| *P. batesiana* | PnTb19 | KP708989 |
| *P. brasiliana* | PnSm07 | KP708990 |
| *P. caciantha* | PnSL05 | KP708992 |
| *P. calliantha* | NWFSC185 | JN091716 |
| *P. circumpora* | PnPd28 | KP708994 |
| *P. cuspidata* | PnPd29 | KP708995 |
| *P. decipiens* | PnKk38 | KP708996 |
| *P. delicatissima* | SZN-B653 | KJ608075 |
| *P. dolorosa* | SZN-B592 | OK103849 |
| *P. fraudulenta* | SZN-B670 | KJ608077 |
| *P. fukuyoi* | PnTb25 | KP708997 |
| *P. galaxiae* | SZN-B606 | KJ608078 |
| *P. galaxiae* | SZN-B617 | KJ608079 |
| *P. granii* | RCC2008 | JN934671 |
| *P. heimii* | NWFSC205 | JN091727 |
| *P. kodamae* | PnPd31 | KP709000 |
| *P. lineola* | NWFSC188 | JN091717 |
| *P. lundholmiae* | PnTb21 | KP709001 |
| *P. mannii* | SZN-B640 | KJ608080 |
| *P. micropora* | PnKk14 | KP709003 |
| *P. multiseries* | NparI | AM235380 |
| *P. multiseries* | Tka2 | U18241 |
| *P. multistriata* | VF2.3 | OK103852 |
| *P. pseudodelicatissima* | SZN-B656 | KJ608082 |
| *P. pungens* | PnKd05 | KP709004 |
| *P. sabit* | PnPd82 | KP709005 |
| *P. subcurvata* | UNC1409 | KX253952 |
| *P. turgidula* | NWFSC220 | FJ222752 |

Table A2, ctd.

| **28 S** | | |
| --- | --- | --- |
| **Species** | **Strain designation** | **GB Accession Number** |
| *Cylindrotheca fusiformis* | UTEX2083 | AF417665 |
| *P. abrensis* | Ner-J2 | KP172231 |
| *P. allochrona* | SZN-B495 | ON775562 |
| *P. allochrona* | SZN-B501 | ON775563 |
| *P. allochrona* | SZN-B507 | KC801041 |
| *P. allochrona* | SZN-B509 | KC801042 |
| *P. americana* | CV2 | U41390 |
| *P. arctica* | RCC2002 | JQ995416 |
| *P. arenysensis* | AL-24 | DQ813811 |
| *P. arenysensis* | SZN-B33 | AF416758 |
| *P. australis* | OM1 | AF417651 |
| *P. batesiana* | PnTb19 | KC147534 |
| *P. bipertita* | Pnmi04 | KR021334 |
| *P. brasiliana* | Brasil 8 | AF469672 |
| *P. calliantha* | AL112 | DQ813841 |
| *P. chiniana* | MC3011 | MN128956 |
| *P. circumpora* | PnSb58 | KC147533 |
| *P. cuspidata* | AL-17 | DQ813809 |
| *P. decipiens* | Mex13 | EF506608 |
| *P. delicatissima* | AL22 | DQ813810 |
| *P. delicatissima* | CV3 | U41391 |
| *P. dolorosa* | AL59 | DQ813813 |
| *P. fraudulenta* | Limens1 | AF417647 |
| *P. fryxelliana* | NWFSC241 | JN050296 |
| *P. fukuyoi* | PnTb25 | KC147535 |
| *P. galaxiae* | Mex23 | AY081136 |
| *P. granii* | RCC2008 | JQ995421 |
| *P. hallegraeffii* | CTD44 2 | MF044022 |
| *P. hallegraeffii* | CTD44 3 | MF044024 |
| *P. hasleana* | NWFSC252 | JN050298 |
| *P. inflatula* | No7 | AF417639 |
| *P. kodamae* | PnPd36 | KF482045 |
| *P. kodamae* | PnPd26 | KF482042 |
| *P. kodamae* | PnPd39 | KF482046 |
| *P. limii* | Pnmi16 | KR021343 |
| *P. linea* | ICMB-156 | FJ489633 |
| *P. lineola* | NWFSC188 | JN050300 |
| *P. lundholmiae* | PnTb10 | KC147538 |
| *P. mannii* | AL101 | DQ813814 |
| *P. micropora* | VPB.B3 | AF417649 |
| *P. multiseries* | NWFSC 011 | AF440772 |
| *P. multistriata* | SZN-B27 | AF416753 |
| *P. nanaoensis* | MC4188 | MG787875 |
| *P. plurisecta* | Ner-A1 | KP172228 |
| *P. pseudodelicatissima* | AL-15 | DQ813808 |
| *P. pungens* | CV4 | U41262 |
| *P. pungens* | NWFSC32 | AF440776 |
| *P. qiana* | MC3007 | MN128952 |
| *P. sabit* | Ps283 | KP288514 |
| *P. seriata* | Nissum3 | AF417652 |
| *P. subcurvata* | CCMP1431 | HQ396851 |
| *P. subfraudulenta* | rensubfrau | AF417646 |
| *P. subpacifica* | RdA8 | AF417642 |
| *P. turgidula* | NWFSC255 | JN050301 |
| *P. turgiduloides* | 124C | EF531709 |
| *P. simulans* | MC281 | MF374774 |

Table A2, ctd.

| **ITS** | | |
| --- | --- | --- |
| **Species** | **Strain designation** | **GB Accession Number** |
| *Fragilariopsis kerguelensis* | 4-20 | EF660061 |
| *P. abrensis* | Ner-J2 | KC409108 |
| *P. allochrona* | SZN-B501 | ON775460 |
| *P. allochrona* | SZN-B509 | ON775456 |
| *P. allochrona* | SZN-B524 | ON775457 |
| *P. allochrona* | SZN-B525 | ON775458 |
| *P. allochrona* | SZN-B853 | ON775459 |
| *P. americana* | Kervel | EU523099 |
| *P. arctica* | P2F2N2 | KT589421 |
| *P. arenysensis* | MexA | DQ329211 |
| *P. arenysensis* | AL-24 | DQ813830 |
| *P. arenysensis* | Ner-D1 | GQ228393 |
| *P. australis* | OEM1 | AY257842 |
| *P. batesiana* | PnTb19 | KC147514 |
| *P. bipertita* | Pnmi04 | KR021318 |
| *P. brasiliana* | Pnkk31 | JN252429 |
| *P. bucculenta* | L1.3 | MH376341 |
| *P. caciantha* | AL-56 | DQ813834 |
| *P. calliantha* | AL-112 | DQ813841 |
| *P. cf. subpacifica* | RdA8 | AY257860 |
| *P. chiniana* | MC3011 | MK411963 |
| *P. circumpora* | PnSb58 | JN252430 |
| *P. cuspidata* | AL-17 | DQ813827 |
| *P. decipiens* | GranCan4-1 | DQ336157 |
| *P. decipiens* | Mex13 | DQ336156 |
| *P. delicatissima* | Tasm 10 | AY257848 |
| *P. delicatissima* | AL-22 | DQ813829 |
| *P. delicatissima* | Laesoe5 | AY257849 |
| *P. dolorosa* | AL-59 | DQ813835 |
| *P. fraudulenta* | NWFSC 200 | FJ222755 |
| *P. fraudulenta* | Limens1 | AY257840 |
| *P. fryxelliana* | NWFSC 241 | JN050288 |
| *P. fukuyoi* | PnTb25 | KC147516 |
| *P. galaxiae* | Mex23 | AY257850 |
| *P. galaxiae* | Sydney4 | DQ336158 |
| *P. granii* | UBC100 | EU051654 |
| *P. hainanensis* | MC3099 | MW042679 |
| *P. hainanensis* | MC3098 | MW042678 |
| *P. hallegraeffii* | CTD44 2 | MF044023 |
| *P. hallegraeffii* | CTD44 3 | MF044025 |
| *P. hasleana* | NWFSC 186 | JN050282 |
| *P. inflatula* | no7 | DQ329204 |
| *P. kodamae* | Pnmi66 | KR021307 |
| *P. kodamae* | Pnmi67 | KR021308 |
| *P. limii* | Pnmi16 | KR021311 |
| *P. lineola* | NWFSC 188 | JN091756 |
| *P. lundholmiae* | PnTb10 | KC147523 |
| *P. mannii* | AL-101 | DQ813839 |
| *P. micropora* | no16 | DQ329209 |
| *P. micropora* | VPB-B3 | AY257847 |
| *P. multiseries* | mu3 | AY257844 |
| *P. multistriata* | KoreaA | AY257843 |
| *P. nanaoensis* | MC4188 | MG787881 |
| *P. obtusa* | T5 | DQ062667 |
| *P. plurisecta* | Ner-F1 | KC409089 |
| *P. pseudodelicatissima* | AL-15 | DQ813826 |
| *P. pungens* | PnMt45 | HQ111412 |
| *P. pungens var. aveirensis* | P-24 | AY257845 |
| *P. qiana* | MC3007 | MK412843 |
| *P. qiana* | MC989 | MK412841 |
| *P. qiana* | MC991 | MK412842 |
| *P. sabit* | PnPd57 | KM400610 |
| *P. seriata* | Nissum3 | AY257841 |
| *P. simulans* | MC281 | MF374769 |
| *P. subcurvata* | 1-F | DQ329205 |
| *P. taiwanensis* | MC5109 | MW042680 |
| *P. turgidula* | NWFSC220 | JN091764 |
| *P. turgiduloides* | 3-19 | AY257839 |

Table A2, ctd.

| ***rbc*L** | | |
| --- | --- | --- |
| **Species** | **Strain designation** | **GB Accession Number** |
| *Cylindrotheca sp.* | N1 | M59080 |
| *P. allochrona* | SZN-B485 | as SZN-B507 |
| *P. allochrona* | SZN-B499 | as SZN-B507 |
| *P. allochrona* | SZN-B501 | as SZN-B507 |
| *P. allochrona* | SZN-B507 | KC801037 |
| *P. allochrona* | SZN-B509 | as SZN-B507 |
| *P. allochrona* | SZN-B524 | as SZN-B507 |
| *P. allochrona* | SZN-B525 | as SZN-B507 |
| *P. allochrona* | SZN-B583 | as SZN-B507 |
| *P. americana* | FBJun06.6 | EF423504 |
| *P. arctica* | RCC2002 | KT808257 |
| *P. arenysensis* | AL64 | DQ813823 |
| *P. arenysensis* | AY11 | EF423516 |
| *P. arenysensis* | SZN-B487 | KC801036 |
| *P. arenysensis* | AL24 | DQ813819 |
| *P. caciantha* | AL56 | DQ813821 |
| *P. calliantha* | Al117 | DQ813825 |
| *P. cuspidata* | AL28 | DQ813820 |
| *P. delicatissima* | AL22 | DQ813818 |
| *P. dolorosa* | AL59 | DQ813822 |
| *P. fraudulenta* | AL75 | EF520333 |
| *P. fraudulenta* | AL50 | EF423502 |
| *P. fraudulenta* | BB19 | EF423503 |
| *P. fryxelliana* | NWFSC 241 | JN050302 |
| *P. galaxiae* | SM3 | EF423512 |
| *P. galaxiae* | AL8 | EF423515 |
| *P. galaxiae* | SM54 | EF423514 |
| *P. hasleana* | NWFSC 186 | JN050304 |
| *P. mannii* | AL101 | DQ813824 |
| *P. multiseries* | NWFSC 316 | KC801040 |
| *P. multistriata* | 279 | EF520337 |
| *P. multistriata* | 19A | EF423505 |
| *P. multistriata* | DD4 | EF520336 |
| *P. pseudodelicatissima* | AL15 | DQ813817 |
| *P. pseudodelicatissima* | SZN-B317 | KC801039 |
| *P. pungens* | Na213 | FM207548 |
| *P. pungens* | FBA2A11 | EF423507 |
| *P. turgiduloides* | 7A1 | EF423508 |
