# Supplementary material for: Phenological segregation suggests speciation by time in the planktonic diatom Pseudo‐nitzschia allochrona sp. nov
Source: Ecol Evol. 2022 Aug 4;12(8):e9155. doi: 10.1002/ece3.9155 (PMC9352866; doi:10.1002/ece3.9155)
Supplement: Supplementary file 3 — Table S3 [file ECE3-12-e9155-s003.docx]

Table A4*:* Results of cross experiments between couples of different strains of *P. allochrona* and between 4 of them and 2 *P. arenysensis* strains of different mating types. Strain names abbreviated retaining only the final part of their name as reported in Table A1. By convention, female sex was attributed to the strains to which zygotes were attached, identified by their size in EM images from crosses between strains with cells of different sizes (Fig. 2 D), and then extrapolated to all strains sexually incompatible with those ones.

| ***P. allochrona*** | | | | | | | | | | | | | | | | | | | ***P. arenysensis*** | |
| --- | --- | --- | --- | --- | --- | --- | --- | --- | --- | --- | --- | --- | --- | --- | --- | --- | --- | --- | --- | --- |
|  | **8-B5**  **♀** | **8-C3**  **♂** | **8-C5**  **♀** | **9-A1**  **♀** | **9-A2**  **♂** | **9-A4**  **♀** | **9-B1**  **♀** | **9-B3**  **♂** | **9-B4**  **♂** | **9-B6**  **♀** | **9-C1**  **♂** | **9-C2**  **♂** | **9-C3**  **♂** | **9-C3A**  **♀** | **9-C4**  **♀** | **9-C5**  **♀** | **9-D3**  **♂** | **9-D4**  **♀** | **BB16**  **♂** | **CM63**  **♀** |
| **8-B5♀** |  | **+ (2)** | **- (2)** | **-** | **+(2)** | **-** | **-** | **+** | **+** | **-** | **+** | **+** | **+** | **-** | **-** | **-** | **+** | **-** | **-** | **-** |
| **8-C3♂** |  |  | **+ (2)** | **+** | **- (2)** | **+** | **+** | **-** | **-** | **+** | **-** | **-** | **-** | **+** | **+** | **+** | **-** | **+** | **-** | **-** |
| **8-C5♀** |  |  |  | **-** | **+(2)** | **-** | **-** | **+** | **+** | **-** | **+** | **+** | **+** | **-** | **-** | **-** | **+** | **-** | **-** | **-** |
| **9-A1♀** |  |  |  |  | **+** | **-** | **-** | **+** | **+** | **-** | **+** | **+** | **+** | **-** | **-** | **-** | **+** | **-** |  |  |
| **9-A2♂** |  |  |  |  |  | **+** | **+** | **-** | **-** | **+** | **-** | **-** | **-** | **+** | **+** | **+** | **-** | **+** | **-** | **-** |
| **9-A4♀** |  |  |  |  |  |  | **-** | **+** | **+** | **-** | **+** | **+** | **+** | **-** | **-** | **-** | **+** | **-** |  |  |
| **9-B1♀** |  |  |  |  |  |  |  | **+** | **+** | **-** | **+** | **+** | **+** | **-** | **-** | **-** | **+** | **-** |  |  |
| **9-B3♂** |  |  |  |  |  |  |  |  | **-** | **+** | **-** | **-** | **-** | **+** | **+** | **+** | **-** | **+** |  |  |
| **9-B4♂** |  |  |  |  |  |  |  |  |  | **+** | **-** | **-** | **-** | **+ (*)** | **+ (*)** | **+ (*)** | **-** | **+** |  |  |
| **9-B6♀** |  |  |  |  |  |  |  |  |  |  | **+** | **+** | **+** | **-** | **-** | **-** | **+** | **-** |  |  |
| **9-C1♂** |  |  |  |  |  |  |  |  |  |  |  | **-** | **-** | **+** | **+** | **+** | **-** | **+** |  |  |
| **9-C2♂** |  |  |  |  |  |  |  |  |  |  |  |  | **-** | **+** | **+** | **+** | **-** | **+** |  |  |
| **9-C3♂** |  |  |  |  |  |  |  |  |  |  |  |  |  | **+** | **+** | **+** | **-** | **+** |  |  |
| **9-C3A♀** |  |  |  |  |  |  |  |  |  |  |  |  |  |  | **-** | **-** | **+ (*)** | **-** |  |  |
| **9-C4♀** |  |  |  |  |  |  |  |  |  |  |  |  |  |  |  | **-** | **+** | **-** |  |  |
| **9-C5♀** |  |  |  |  |  |  |  |  |  |  |  |  |  |  |  |  | **+** | **-** |  |  |
| **9-D3♂** |  |  |  |  |  |  |  |  |  |  |  |  |  |  |  |  |  | **+** |  |  |
| **9-D4♀** |  |  |  |  |  |  |  |  |  |  |  |  |  |  |  |  |  |  |  |  |
| **BB16♂** |  |  |  |  |  |  |  |  |  |  |  |  |  |  |  |  |  |  |  | **+** |
| **CM63♀** |  |  |  |  |  |  |  |  |  |  |  |  |  |  |  |  |  |  |  |  |

**+**: sexual reproduction observed

**–**: sexual reproduction not observed

(2): replicated crosses

*: crosses used for electron microscopy observations

♀: female mating types

♂: male mating types

dark grey boxes: crosses not performed
