# Supplementary material for: Phenological segregation suggests speciation by time in the planktonic diatom Pseudo‐nitzschia allochrona sp. nov
Source: Ecol Evol. 2022 Aug 4;12(8):e9155. doi: 10.1002/ece3.9155 (PMC9352866; doi:10.1002/ece3.9155)
Supplement: Supplementary file 4 — Table S4 [file ECE3-12-e9155-s001.docx]

Table A5: Estimates of net evolutionary divergence between species of the *P. delicatissima*-complex closest to *P. allochrona*, using a Maximum Composite Likelihood model. Standard error estimates (italics, above the diagonal) were estimated through 100 bootstrap replicates.

|  | *P. allochrona* | *P. arenysensis* | *P. delicatissima* | *P. dolorosa* | *P. decipiens* | *P. micropora* |
| --- | --- | --- | --- | --- | --- | --- |
| *P. allochrona* |  | *0.010* | *0.011* | *0.028* | *0.023* | *0.013* |
| *P. arenysensis* | 0.042 |  | *0.012* | *0.030* | *0.023* | *0.009* |
| *P. delicatissima* | 0.090 | 0.090 |  | *0.026* | *0.019* | *0.007* |
| *P. dolorosa* | 0.251 | 0.283 | 0.246 |  | *0.025* | *0.025* |
| *P. decipiens* | 0.189 | 0.205 | 0.163 | 0.258 |  | *0.016* |
| *P. micropora* | 0.082 | 0.045 | 0.024 | 0.234 | 0.133 |  |
