# Supplementary material for: Phenological segregation suggests speciation by time in the planktonic diatom Pseudo‐nitzschia allochrona sp. nov
Source: Ecol Evol. 2022 Aug 4;12(8):e9155. doi: 10.1002/ece3.9155 (PMC9352866; doi:10.1002/ece3.9155)
Supplement: Supplementary file 5 — Figure S1 [file ECE3-12-e9155-s004.pptx]

## Slide 1
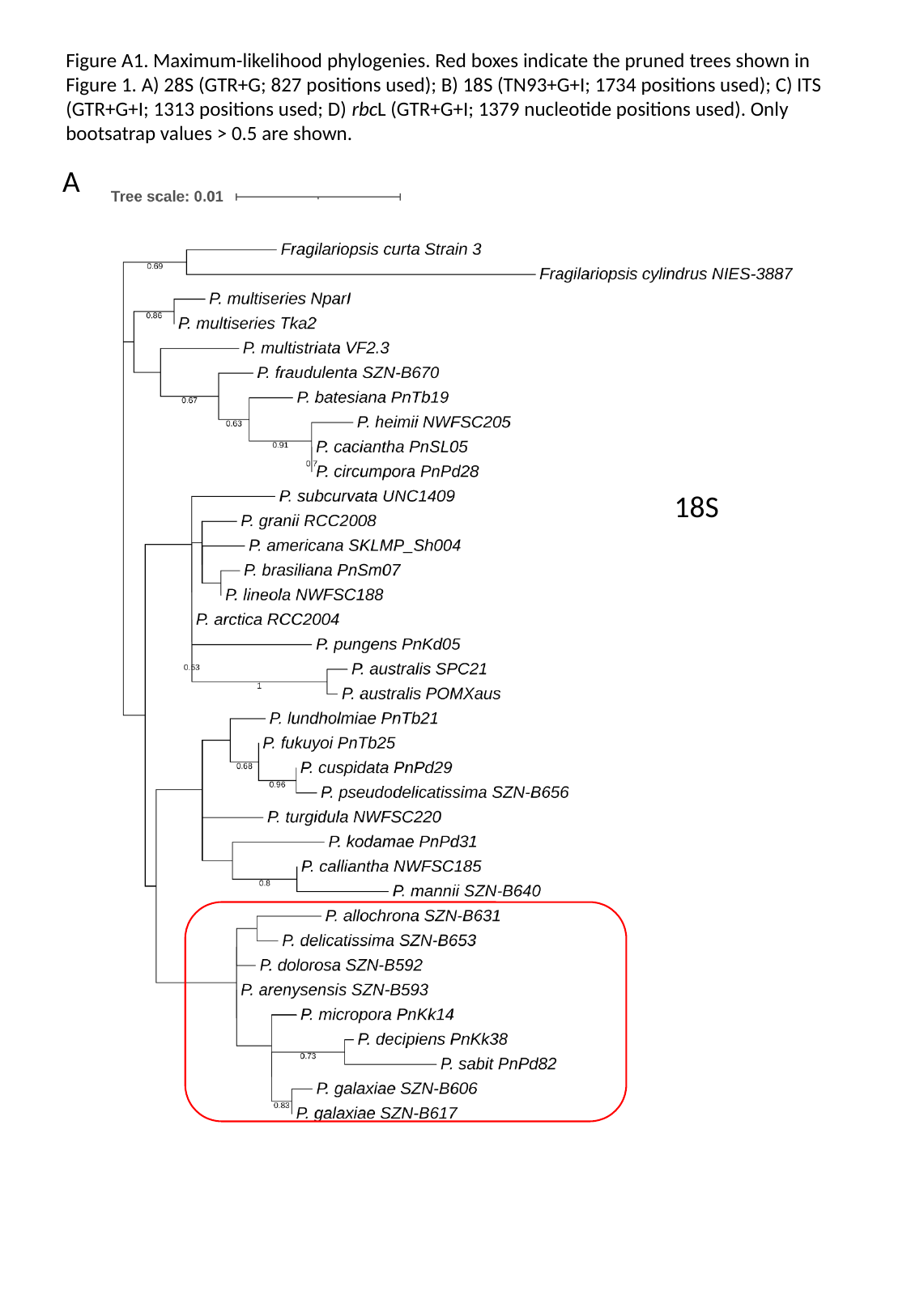

Figure A1. Maximum-likelihood phylogenies. Red boxes indicate the pruned trees shown in Figure 1. A) 28S (GTR+G; 827 positions used); B) 18S (TN93+G+I; 1734 positions used); C) ITS (GTR+G+I; 1313 positions used; D) rbcL (GTR+G+I; 1379 nucleotide positions used). Only bootsatrap values > 0.5 are shown.
A
18S

## Slide 2
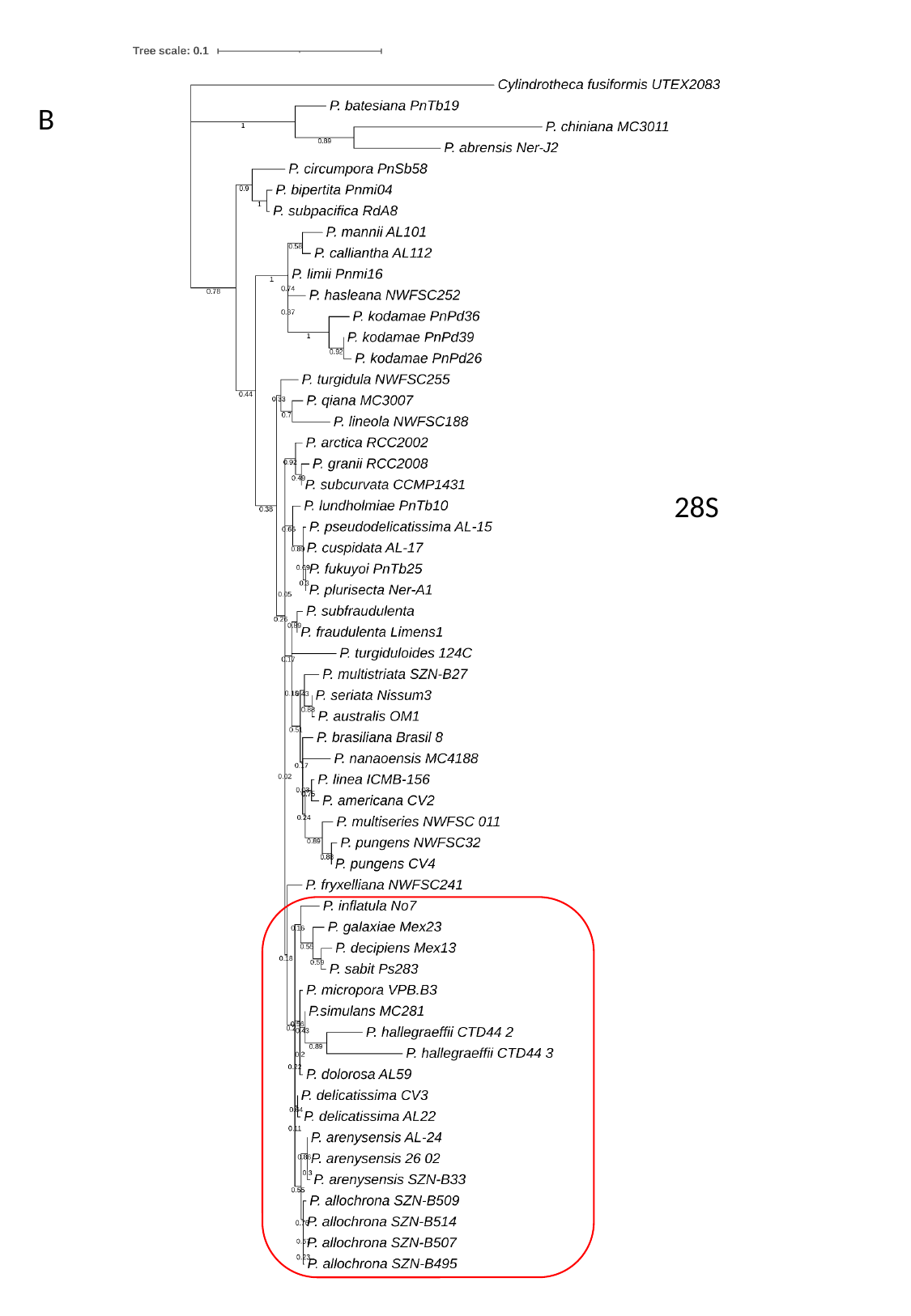

B
28S

## Slide 3
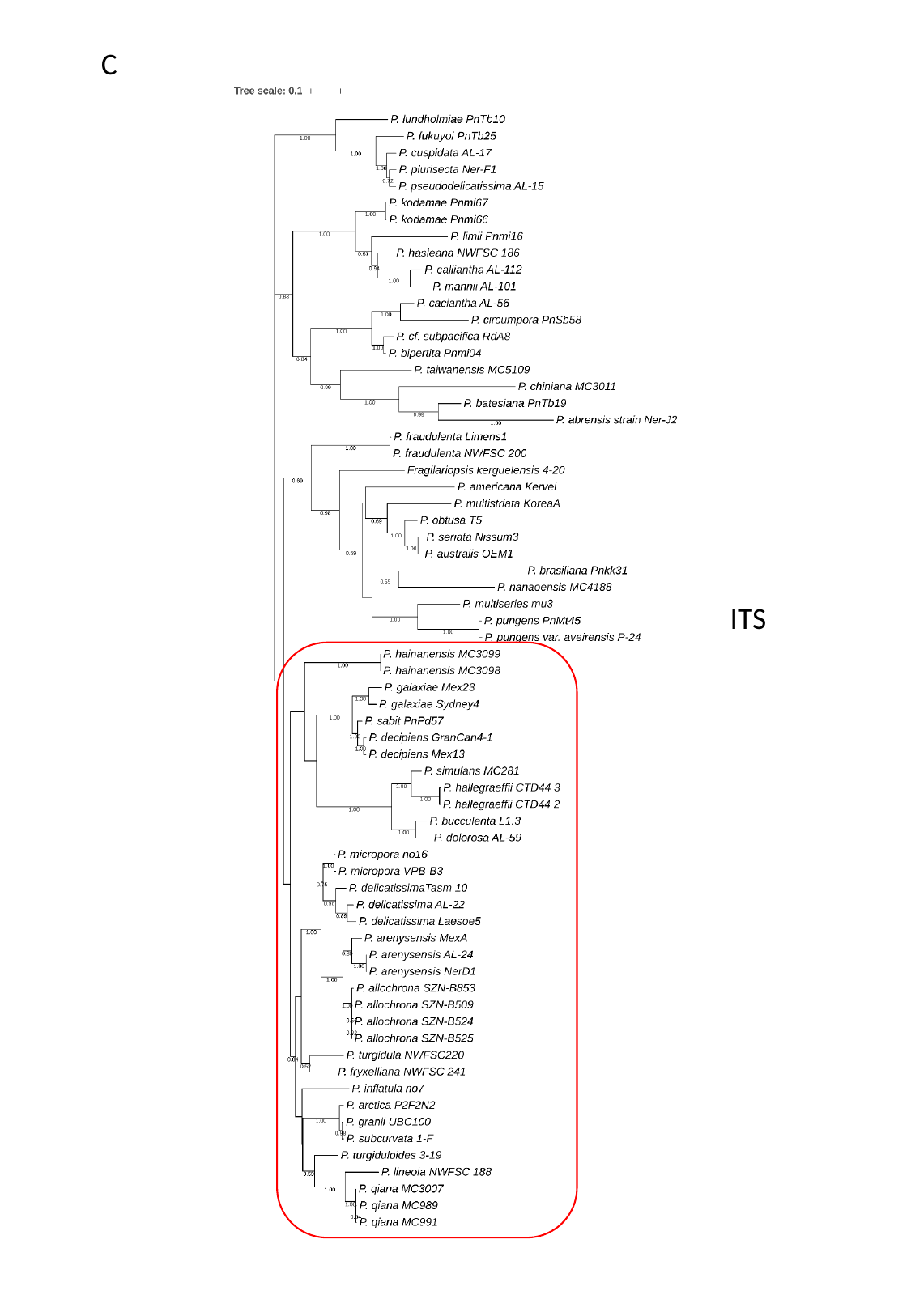

C
ITS

## Slide 4
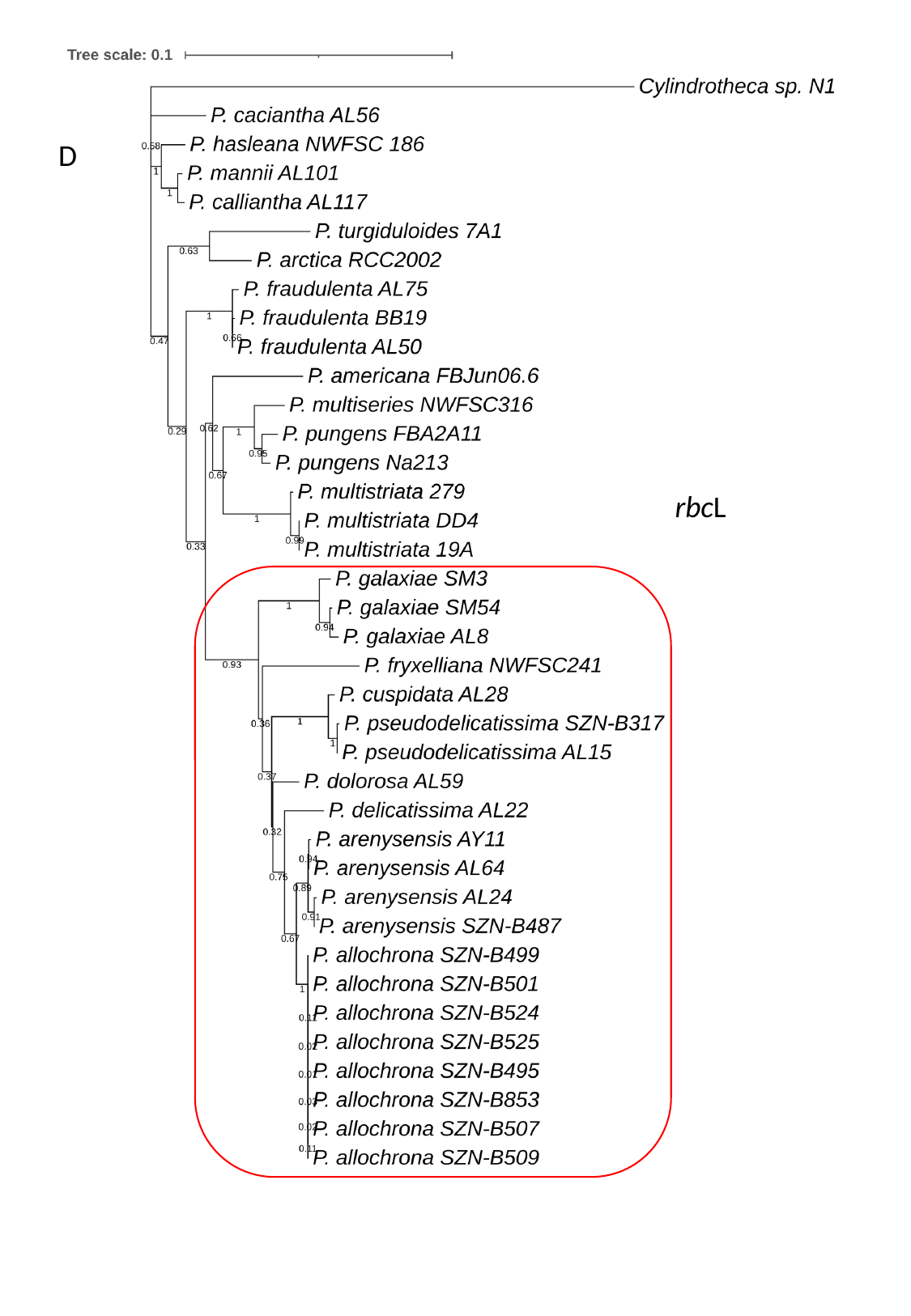

D
rbcL
